# Peroxiredoxin 1 interacts with and blocks the redox factor APE1 from activating interleukin-8 expression

**DOI:** 10.1038/srep29389

**Published:** 2016-07-08

**Authors:** Hassan Nassour, Zhiqiang Wang, Amine Saad, Arturo Papaluca, Nicolas Brosseau, El Bachir Affar, Moulay A. Alaoui-Jamali, Dindial Ramotar

**Affiliations:** 1 Maisonneuve-Rosemont Hospital, Research Center, Université de Montréal, Department of Medicine, 5415 Boul. de l’ Assomption, Montréal, Québec, H1T 2M4, Canada; 2 Segal Cancer Centre and Lady Davis Institute for Medical Research, Sir Mortimer B. Davis-Jewish General Hospital, 3755 Cote Ste-Catherine Road, Montréal, Québec, H3T 1E2, Canada

## Abstract

APE1 is an essential DNA repair protein that also possesses the ability to regulate transcription. It has a unique cysteine residue C65, which maintains the reduce state of several transcriptional activators such as NF-κB. How APE1 is being recruited to execute the various biological functions remains unknown. Herein, we show that APE1 interacts with a novel partner PRDX1, a peroxidase that can also prevent oxidative damage to proteins by serving as a chaperone. PRDX1 knockdown did not interfere with APE1 expression level or its DNA repair activities. However, PRDX1 knockdown greatly facilitates APE1 detection within the nucleus by indirect immunofluorescence analysis, even though APE1 level was unchanged. The loss of APE1 interaction with PRDX1 promotes APE1 redox function to activate binding of the transcription factor NF-κB onto the promoter of a target gene, the proinflammatory chemokine IL-8 involved in cancer invasion and metastasis, resulting in its upregulation. Depletion of APE1 blocked the upregulation of IL-8 in the PRDX1 knockdown cells. Our findings suggest that the interaction of PRDX1 with APE1 represents a novel anti-inflammatory function of PRDX1, whereby the association safeguards APE1 from reducing transcription factors and activating superfluous gene expression, which otherwise could trigger cancer invasion and metastasis.

Apurinic/apyrimidinic (AP) endonuclease1/redox factor-1 (APE1/Ref-1) is a multifunctional protein involved in the base excision repair (BER) of damaged DNA, as well as in transcriptional regulation^1^. These functions reside within distinct domains of the protein (Fig. 1a). APE1 hydrolyzes the 5′-phosphodiester bond at AP sites and removes a variety of blocked 3′ termini at DNA strand breaks with the aid of an AP endonuclease, 3′-diesterase and 3′- to 5′-exonuclease in order to facilitate DNA repair synthesis^1^,^2^,^3^,^4^. Besides its DNA repair activities, APE1 directly or indirectly regulates transcription^1^. For example, APE1 can form a complex with p300 and bind to the calcium responsive elements to suppress gene expression^5^. Furthermore, APE1 can influence the DNA binding activity of various transcription factors such as AP-1^6^, NF-κB^7^, Myb^8^, p53^9^, hypoxia inducible factor-1^10^ and Pax proteins^11^ via its redox cysteine residue C65 by reducing these transcription factors to ensuring their binding onto the promoter of target genes. A recent study has also shown that APE1 can negatively regulate the function of the nuclear factor erythroid-related factor 2 (NRF2), which plays a role in the defense against oxidative stress^12^. Inhibition of the redox function of APE1 potently activates NRF2 target genes, but in a manner that is independent of the production of reactive oxygen species (ROS)^12^.

In order for APE1 to execute its role in DNA repair and gene regulation, there must be regulatory mechanisms that switch on/off- and fine-tune the different APE1 activities and these include (i) alteration in APE1 redox state^13^, (ii) translocation of APE1 from the cytoplasm to the nucleus^14^, and (iii) modulation of APE1 by post-translational modification (PTMs)^5^,^15^,^16^ and proteolytic cleavage of the N-terminal 33 amino acid domain^17^. Besides these mechanisms, APE1 is known to exist in complexes with other proteins and thus modulation of its partners within the interactome could also influence APE1 function. Approximately thirty proteins have been discovered to interact with APE1 using different approaches, however, the functional implications of APE1 interaction with each individual protein partner is still not known^18^,^19^. So far, a single tag, such as HA fused to APE1, has been used to identify APE1 interacting partners^18^. The disadvantage of the single tag approach is that it brings along some non-specific proteins. Moreover, most of the studies performed to identify proteins that interact with APE1 were done under physiological conditions. In this study, we used a stringent tandem affinity approach to investigate the APE1 interactome under physiological conditions and when the cells were challenged with the oxidant hydrogen peroxide (H_2_O_2_). Herein, we discovered a novel APE1 interacting partner, PRDX1, which is a member of the peroxiredoxin family that acts as a peroxidase as well as serving as a chaperone to protect proteins from oxidative damage. PRDX1 interacted with APE1 under physiological conditions, both in the nucleus and cytosol. We show that shRNA knockdown of PRDX1 has no effect on APE1 expression level or its DNA repair activities. However, indirect immunofluorescence revealed that the knockdown of PRDX1 amplified the detection of APE1 in the nucleus, even though APE1 level was not altered. While this observation is consistent with APE1-PRDX1 association *in vivo*, it raises the possibility that PRDX1 might influence the transcriptional function of APE1. Indeed, we show that PRDX1 knockdown enhanced APE1 redox activity, and which acts via the transcription factor NF-κB to turn on expression of the proinflammatory chemokine interleukin-8 (IL-8) that has been shown to play a role in cancer invasion and metastasis^20^,^21^. In view of the regulatory role of APE1-PRDX1 interaction, we propose that PRDX1 through its association with APE1 keeps a check on APE1 redox activity from reducing and activating the transcription factor NF-κB, and thereby prevents superfluous gene expression of IL-8. To the best of our knowledge, our study is the first to demonstrate a novel role of PRDX1 as an anti-inflammatory protein that functions by blocking APE1 from activating IL-8.

## Results

### Expression and purification of APE1 interacting complex from HeLaS cells

**Figure 1:**
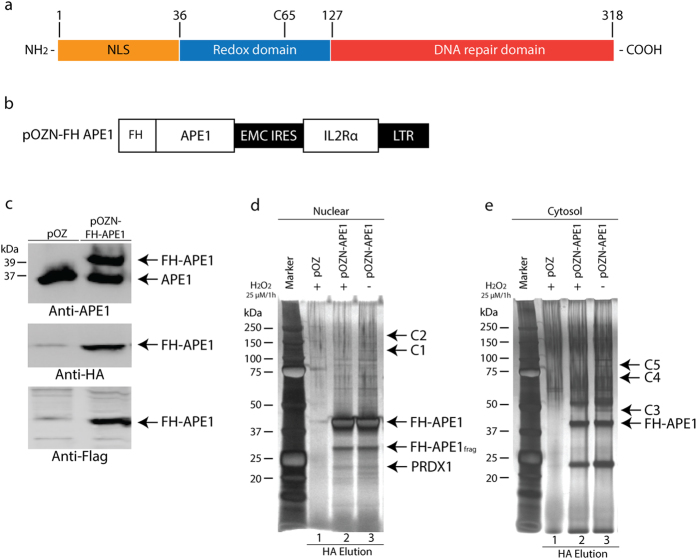
Structural features of APE1 and expression of the tagged form, FH-APE1, used for the complex purification from HeLaS cells. (**a**) Illustration of the structural domains of APE1. **(b**) Schematic representation of FH-APE1 construct in which IL2Rα is used for selection. (**c**) Western blot analysis validating the ectopic FH-APE1 expression. HeLaS cells were infected with retroviruses containing either the empty vector pOZ or the plasmid pOZN-FH-APE1, followed by three rounds of selection with anti-IL2Rα magnetic beads and positive cells were expanded. Total cell extracts were analyzed by Western blot probed with monoclonal anti-APE1, anti-FLAG and anti-HA respectively. (**d**,**e**) APE1 complex following purification from nuclear and cytosolic extracts, respectively. HeLaS cells expressing FH-APE1 were untreated or treated with 25 μM H_2_O_2_ for 1 h, while the HeLaS cells containing empty vector pOZ were only treated with 25 μM H_2_O_2_ for 1 h and serve as negative control for subsequent immunoprecipitation. The cells were harvested and the nuclear and cytosolic fractions were subjected to tandem immunoprecipitation with anti-FLAG followed by anti-HA resins. APE1 complex were finally eluted by HA peptides and separated on 4 to 12% gradient SDS-PAGE gels followed by silver staining. Pooled eluates were subjected to mass spectrometry to identify all the proteins forming part of the APE1 interactome. C1 and C2, and C3 to C5, indicate polypeptide bands that disappeared from the nuclear and cytosolic APE1 complex, respectively, in response to the H_2_O_2_ treatment. FH-APE1_frag_ denotes a proteolytic form of FH-APE1. The data are representative of two independent complex purifications.

Previously, a single affinity tag was used to explore APE1 interacting partners from total cell extracts^22^. In this work, we examined the APE1 interacting partners under more stringent purification conditions by employing two affinity purification tags, FLAG and HA. In addition, we used this approach to determine whether the APE1 interactome would be influenced upon exposing cells to oxidative stress caused by the oxidant H_2_O_2_. We created stable HeLaS cell lines expressing a N-terminal FLAG-HA tagged APE1 from the pOZ vector (Fig. 1b) (referred herein as FH-APE1)^23^. The ectopic expression of FH-APE1 in HeLaS was validated by Western blot probed with three different antibodies, anti-APE1, anti-HA and anti-FLAG (Fig. 1c). The pOZ vector expressed FH-APE1 with the expected molecular weight of 39 kDa and to nearly the same level as the endogenous APE1 (37 kDa)(upper panel Fig. 1c).

We used this expression system to examine the proteins associated with FH-APE1 in nuclear and cytosolic extracts of the HeLaS cells. The FH-APE1 complex from nuclear extracts was purified by tandem affinity purification using anti-FLAG and -HA resins. The final HA elutions were concentrated by trichloroacetic acid (TCA) and an aliquot analyzed by SDS-PAGE that was stained with silver (Fig. 1d). Instead of isolating individual bands from the SDS-PAGE, the entire HA eluate was subjected to mass spectrometry in order to identify all the proteins. HA eluate from nuclear extract carrying the pOZ empty vector was used as a control. This stringent approach identified only five proteins (APE1, LMNA, NPM1, PRDX1 and RPS19) from the nuclear fraction and each protein was considered a hit after setting a limit for three unique peptides (Fig. 1d and Supplementary Table S2). Amongst these proteins only NPM1 was previously reported to interact with APE1 using a single affinity purification^18^. LMNA, PRDX1 and RPS19 were found for the first time to be part of the APE1 interactome. In fact, the STRING database of known and predicted protein interactions suggested a connection between NPM1 and PRDX1 on the basis that PRDX1 interacts with some of the same proteins found to interact with FH-APE1 (Supplementary Fig. S1A)^24^.

The same approach was used to identify FH-APE1 complex in the cytosol (Fig. 1e). FH-APE1 was found to interact with 14 proteins (Supplementary Fig. S1b and Supplementary Table S2) and four of these PRDX1, PDIA6, PRDX2, PRDX3 are known to play a direct role in mitigating oxidative stress^25^,^26^,^27^,^28^ (Supplementary Table S2). In fact, the STRING database suggested a connection between HSP90AB1 and APE1 on the basis that HSP90AB1 interacts with partners of FH-APE1 (Supplementary Fig. S1b)^24^. These findings suggest that APE1 forms distinct complexes in the nucleus and the cytoplasm and it appears that in the cytosol APE1 forms complexes with proteins that are involved in oxidative stress responses.

### APE1 interactome changes upon treatment with H_2_O_2_

We next examined whether APE1 interaction with its partners could be influenced by oxidative stress. The HeLaS cells expressing FH-APE1 were untreated and treated with H_2_O_2_ and the proteins associated with APE1 in the nucleus were determined by mass spectrometry. Upon treatment with a very mild dose of H_2_O_2_ (25 μM for 1 h), that killed less than 7% of the cells, there was no major change in the affinity purified complex, except for at least two polypeptides indicated as C1 and C2 that were diminished (Fig. 1d). However, using four fold higher concentration (100 μM for 1 h) resulted in decreased cell viability by 40%, with concomitant loss of all the nuclear APE1 interacting partners, with the exception of LMNA.

We used the same approach to examine the potential APE1 interacting partners in the cytosol upon H_2_O_2_ treatment. There was a decrease in the number of proteins that were associated with APE1 under the H_2_O_2_ treatment condition (25 μM for 1 h) and denoted as C3, C4 and C5, as compared to the untreated counterpart (Fig. 1e, lane 3 vs. 2). The oxidative stress did not interfere with some of the APE1 interactions, such as PRDX1 and PDIA6 that co-existed with APE1 under physiological conditions, but other interactions were lost and some new ones appeared (Supplementary Fig. S1b vs. S1c, Supplementary Table S2). At least nine new interactions with APE1 were detected in the cytosol following the H_2_O_2_ treatment (25 μM for 1 h) (Supplementary Fig. S1c). Importantly, H_2_O_2_ treatment preserved the interaction of APE1 with proteins involved in oxidative stress responses such as PRDX1, PDIA6, as well as gained an interaction with PRDX6 (Supplementary Fig. S1b vs. S1c). These data suggest that APE1 interaction with its partners can be regulated by oxidative stress.

### Validation of interaction between APE1 and PRDX1

**Figure 2:**
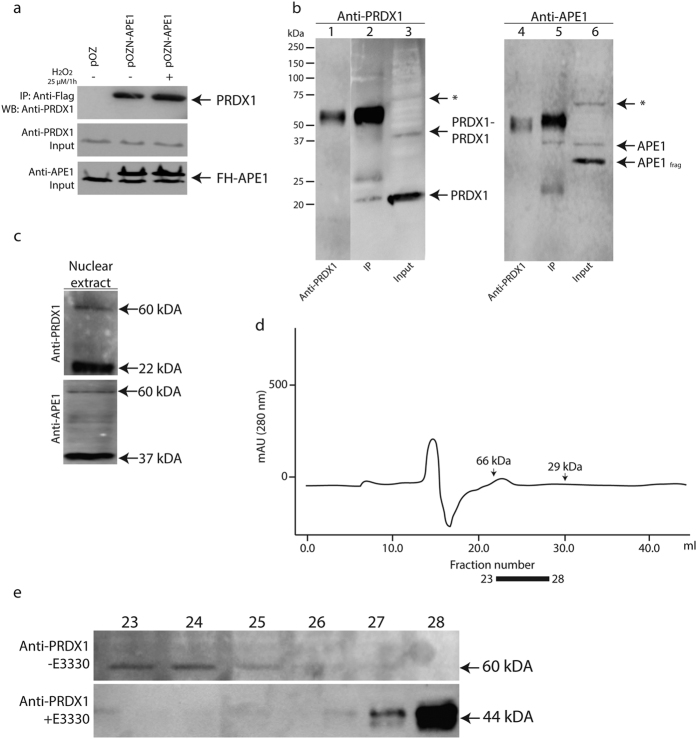
Validation of APE1-Prdx1 interaction. (**a**) FH-APE1 pulled down Prdx1 during immunoprecipitation. HeLaS cells expressing FH-APE1 were untreated or treated with 25 μM H_2_O_2_ for 1 h, while the control HeLaS cells containing empty vector pOZ were untreated, and total cell extracts were subjected to immunoprecipitation with anti-FLAG resins. Eluates were separated by 10% SDS-PAGE and probed with monoclonal anti-PRDX1. (**b**) Reciprocal immunoprecipitation showing that PRDX1 pulls down APE1 from nuclear extract. Nuclear extract prepared from HeLa was subjected to immunoprecipitation with protein A magnetic beads bearing anti-PRDX1 antibodies. Lanes 2 and 5, immunoprecipitated proteins, lanes 3 and 6, the input nuclear extract, and lanes 1 and 4, each contained 0.5 μg of anti-PRDX1 antibodies alone. Following 10% SDS-PAGE for immunoblot analysis, lanes 1 to 3 were probed with anti-PRDX1 antibodies and lanes 4 to 6 with anti-APE1 antibodies. The arrows marked by an asterisk indicate a similar size polypeptide ~60 kDa detected by either anti-PRDX1 or anti-APE1 antibodies. (**c**) Detection of a 60 kDa polypeptide by either anti-PRDX1 or anti-APE1 antibodies in nuclear extract. Nuclear extract from HeLa cells prepared in the present of the thiol blocking agent methyl methanethiosulfonate (MMTS) was subjected to Western blot analysis probed with either anti-PRDX1 or anti-APE1 antibodies. (**d**) Analysis of APEX1-PRDX1 conjugate by fast protein liquid chromatography (FPLC). HeLa cells were untreated or treated with E3330 and the nuclear extract was loaded on superose^TM^ 6 FPLC column eluted with buffer A at flow rate of 0.25 ml/min. All eluents were collected at 0.5 ml/fraction, and each eluent fraction was concentrated by Amicon Ultra followed by Western blot analysis with 10% SDS PAGE. (**e**) The APE1 specific inhibitor E3330 prevents the appearance of the 60 kDa polypeptide and causes the accumulation of the dimeric PRDX1. Eluent fractions 23 to 28 from E3330 untreated and treated cells were probed with monoclonal anti-PRDX1. Fractions 23 and 24 contained the 60 kDa polypeptide from E3330 untreated cells, while fraction 28 contained the dimeric PRDX1 (44 kDa) from E3330 treated cells. The data are representative of two or three independent experiments.

Under physiological conditions, PRDX1 was the only common partner that existed with APE1 in both the nucleus and the cytosol. PRDX1 has been documented to function as an antioxidant enzyme that scavenges H_2_O_2_^29^,^30^. PRDX1 thus protects proteins, lipids, and hormone receptors from oxidation-induced inactivation and inhibits tumorigenesis^31^,^32^. Recent studies demonstrated that mice deficient in PRDX1 caused tissue specific loss of heterozygosity implying that PRDX1 may be involved in maintaining genomic stability perhaps through its interaction with APE1^33^. To test whether APE1 and PRDX1 indeed belong to a complex, we carried out co-immunoprecipitation experiments with anti-FLAG resin using total protein extracts derived from HeLaS cells carrying either the empty pOZ vector as control or pOZN-FH-APE1 under conditions where the cells were untreated or treated with H_2_O_2_ (25 μM for 1 h). The anti-FLAG antibodies pull down PRDX1 from total extracts expressing FH-APE1, but not from extracts carrying the empty vector (Fig. 2a). The co-immunoprecipitation of PRDX1 with FH-APE1 was not affected when the cells were treated with the low concentration of H_2_O_2_ (Fig. 2a), consistent with the mass spectrometry data.

We conducted the reciprocal experiment by using protein A magnetic beads attached with anti-PRDX1 monoclonal antibodies to immunoprecipitate PRDX1 from nuclear extract derived from HeLa cells and examined for the pull down of APE1. The nuclear extract contained the monomeric PRDX1 (22 kDa), the dimeric form (44 kDa) and perhaps a weaker high molecular weight form (~60 kDa, shown by an asterisk) (Fig. 2b, lane 3), as detected by anti-PRDX1. In addition, the nuclear extract contained the native APE1 (37 kDa), a fragment of APE1 (APE1_frag_ ~32 kDa) and a higher molecular weight form (~60 kDa, shown by an asterisk) (Fig. 2b, lane 6), as detected by anti-APE1. The anti-PRDX1 magnetic beads pulled down the monomeric PRDX1, but not the dimeric form of PRDX1 or the weak 60 kDa polypeptide, as detected by the anti-PRDX1 antibodies (Fig. 2b, lane 2). The additional bands present in lane 2 were the light and heavy chains of the anti-PRDX1 antibodies, as confirmed by loading only a small amount of anti-PRDX1 on the gel (lane 1). Importantly, the anti-PRDX1 magnetic beads pulled down only the native APE1 (the 37 kDa band in lane 5), and not the APE1_frag_ (~32 kDa) or the ~60 kDa form, as detected by anti-APE1 antibodies (Fig. 2b, lane 5), consistent with APE1 interacting with PRDX1.

To further validate the interaction between APE1 and PRDX1, we examined for formation of APE1-PRDX1 conjugates *in vivo* by preparing nuclear extract in the presence of the thiol blocking agent methyl methanethiosulfonate (MMTS) to prevent reversal of the complex and subjecting the extract to gel filtration chromatography using FPLC. Under this condition, the nuclear extract contained a prominent 60 kDa polypeptide that was detected by both anti-APE1 and anti-PRDX1 antibodies (Fig. 2c), suggesting that the polypeptide contains both APE1 and PRDX1. Upon gel filtration analysis, the 60 kDa polypeptide co-eluted with the molecular weight standard albumin (66 kDa) and eluted in fractions 23 and 24 (Fig. 2d,e). Treatment of the cells with (E)-3-[2-(5,6-dimethoxy-3-methyl-1,4-benzoquinonyl)]-2-nonyl propenoic acid (E3330), a specific inhibitor that inhibits the redox activity of APE1 through Cys-65, Cys-93 and Cys-99 oxidation^34^,^35^, caused the disappearance of the 60 kDa polypeptide and the appearance of a very prominent 44 kDa protein, similar in size as the dimeric PRDX1, that was detected by anti-PRDX1 in fraction 28 (Fig. 2e). Taken together, these data are consistent with the immunoprecipitation results (Fig. 2a,b) that PRDX1 interacts with APE1 to form a heterodimeric complex of nearly 60 kDa in size. Moreover, our data further suggest that in the absence of APE1 redox function, PRDX1 may exist in the oxidized (44 kDa) dimeric form.

### Prdx1 knockdown did not affect APE1 protein abundance or gene expression

**Figure 3:**
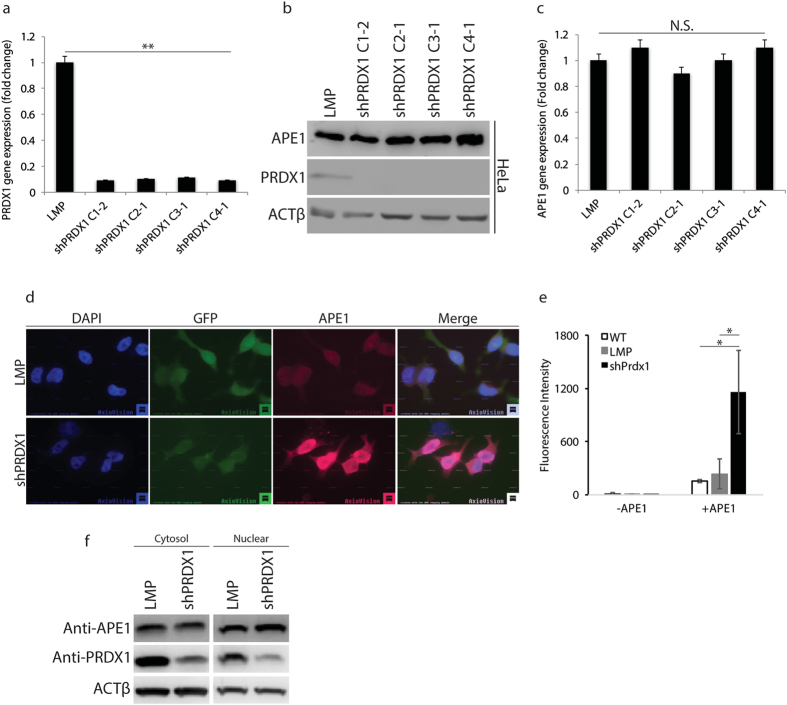
PRDX1 knockdown augments the detection of APE1 in the nucleus. Four different shRNA (C1-2, C2-1, C3-1, and C4-1) against PRDX1 and the LMP vector (control) packaged into retroviruses were used for infecting HeLa cells to create stable clones. After one week of selection with 0.5 μg/ml puromycin, the cells were analyzed for the expression levels of the indicated genes and proteins. (**a**) shPRDX1 downregulates *PRDX1* gene in HeLa cells. The expression of *PRDX1* was monitored by quantitative PCR analysis using total RNA isolated from HeLa cells carrying either the control LMP vector or the different shPRDX1 constructs. *PRDX1* expression was normalized to the *ACTβ* gene and the levels in the shPRDX1 knockdown cells were expressed as fold-change relative to the level in the LMP control, which was set at 1.0. (**b**) Western blot analysis showing APE1 and PRDX1 protein levels in the shPRDX1 knockdown HeLa cells. The Western blot was first probed with anti-PRDX1 antibodies and then for the levels of APE1 and *ACTβ* using anti-APE1 and anti- ACTβ antibodies, respectively. (**c**) Expression level of the *APE1* gene in the shPRDX1 knockdown cells. APE1 expression was monitored by qPCR as in panel **a**. (**d**) Indirect immunofluorescent analysis of HeLa-LMP and HeLa-shPRDX1 cells stained with polyclonal anti-APE1 antibodies. Images were captured with a Zeiss epifluorescent microscope using Cy5 and DAPI-UV filters at 67x magnification and processed with ImageJ. Merged images overlapped DAPI-stained nucleus (blue) with Cy5 staining of APE1 (red). FITC filter marks the cells expressing the GFP protein from the LMP vector. (**e**) Detection of APE1 by flow cytometry. The indicated cells were incubated without and with anti-APE1 antibodies and the APE1 signal was detected by Alexa Fluor^®^ 594 with the FL2 (585/42) channel using FACS calibur. (**f**) APE1 subcellular distribution in the control and PRDX1 knockdown HeLa cells. APE1 and PRDX1 levels in the cytosol and nucleus of the indicated cells were determined with antibodies against APE1 and PRDX1 relative to the loading control ACTβ. The data are representative of two or three independent experiments.

To explore the role of PRDX1 on APE1 function, we knocked down PRDX1 in two different cell lines, HeLa and HepG2, via a retroviral system to express shRNA against PRDX1. Briefly, the shRNA-PRDX1 (shPRDX1) or its vector control LMP transfected cells were selected for one week in the presence of puromycin (0.5 ug/ml), harvested and examined for *PRDX1* gene expression level by quantitative qPCR and the protein level by Western blot analysis using anti-PRDX1 monoclonal antibody. Four shRNA that targeted different regions of *PRDX1*, all decreased *PRDX1* gene expression level and as well as the protein level by nearly 90% in the HeLa cells, as compared to the control LMP vector and where ACTβ was used as a control (Fig. 3a,b, respectively). One of these knockdowns, shPRDX1 C1-2 was chosen for the rest of the studies, and this construct was also effective in downregulating PRDX1 in another cell line, namely HepG2 (Supplementary Fig. S2a). While PRDX1 level was decreased in the shPRDX1 knockdown cells, the protein level of APE1 remain unchanged in these cells as determined by Western blot probed with anti-APE1 antibody (Fig. 3b and Supplementary Fig. S2a). Consistent with the Western blot analysis, *APE1* gene expression was also unaltered in the different shPRDX1 knockdown clones (Fig. 3c), indicating that PRDX1 is not involved in regulating the expression level of APE1.

### Prdx1 knockdown enhances APE1 detection in the nucleus

Since PRDX1 was found associated with APE1 in both the cytosol and the nucleus, we checked whether PRDX1 knockdown would alter APE1 cellular distribution. We performed indirect immunofluorescence using a polyclonal antibody against APE1 and found that APE1 detection in the nucleus was much more intense in the shPRDX1 HeLa cells, as opposed to the control cells carrying the empty LMP vector (Fig. 3d). A similar result was obtained with monoclonal antibody against APE1 (Supplementary Fig. S3). For these experiments, we could not simultaneously monitor the detection of PRDX1 in the LMP control *versus* the shPRDX1 HeLa cells as there are no commercially available anti-PRDX1 antibodies suitable for immunofluorescence analysis.

To independently check that there was indeed enhanced detection of APE1 in the shPRDX1 HeLa cells, we monitored for APE1 signal in the LMP control and shPRDX1 HeLa cells by FACS Calibur using Alexa Fluor^®^ 594 as the secondary antibody to detect anti-APE1. In the absence of the anti-APE1 antibodies, no APE1 signal was detected from any of the three cell lines HeLa alone, HeLa with the control LMP vector or shPRDX1 (Fig. 3e). When anti-APE1 was present, the APE1 signal was detected to the same extent in both the HeLa cells and the cells carrying the LMP vector (Fig. 3e). In contrast, the APE1 signal was greatly enhanced by nearly 5-fold in the shPRDX1 HeLa cells (Fig. 3e). Clearly, the result from the FACS analysis (Fig. 3e) is consistent with that from the immunofluorescence analysis (Fig. 3d).

To exclude the possibility that the increased detection of APE1 in the PRDX1 knockdown cells could be due to mobilization of undetected forms of APE1 within the cell that translocate to the nucleus, we examined the cytosolic and nuclear levels of APE1 in the control and PRDX1 knockdown cells. There was no major change in the level of the cytosolic and nuclear levels of APE1 in the PRDX1 knockdown cells as compared to the LMP vector control (Fig. 3f). Since the total amount of APE1 in the cells did not change (Fig. 3b) and there was no significant increase in the level of APE1 in the nucleus when PRDX1 was downregulated (Fig. 3f), then mobilization of APE1 from other cellular compartments to the nucleus cannot account for the greatly enhanced detection of nuclear APE1 in the PRDX1 knockdown cells (Fig. 3d,e). On the basis of these findings, we raised the possibility that PRDX1 association with APE1 *in vivo* may prevent anti-APE1 antibodies from properly recognizing the epitopes on APE1. While this interpretation is consistent with PRDX1 forming a complex with APE1, it also raises the possibility that PRDX1 could be involved in regulating APE1 function.

### PRDX1 knockdown did not affect APE1 ability to repair damaged DNA

**Figure 4:**
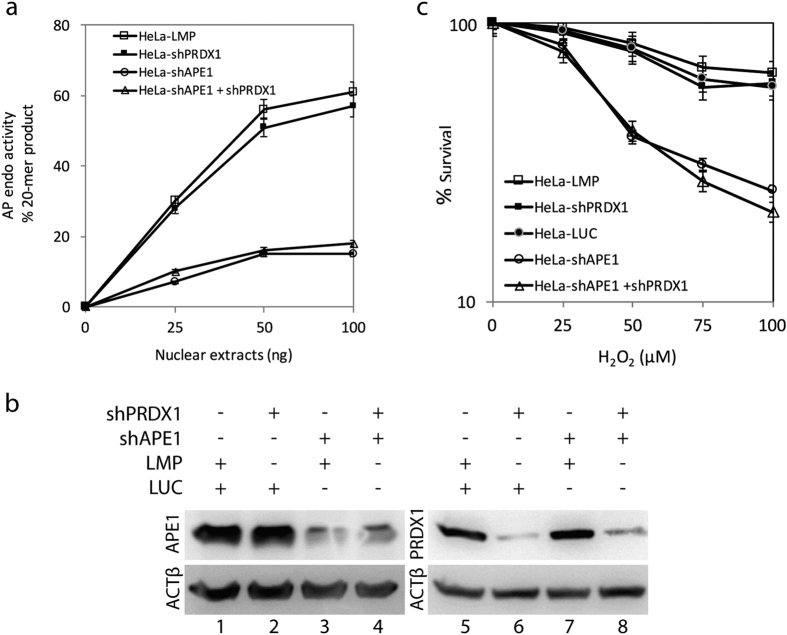
PRDX1 knockdown did not interfere with APE1 ability to repair damaged DNA. (**a**) Total nuclear extracts derived from the four indicated HeLa cell lines were assayed for APE1 AP endonuclease activity using a 42-mer double stranded oligonucleotide substrate carrying a centrally located AP site. Cleavage of the substrate produced a 20-mer product that was quantified and plotted against the concentration of the nuclear extracts. The AP endonuclease activity measurement is the average of two experiments. (**b**) Western blot showing that APE1 knockdown did not alter PRDX1 level or vice versa. Total cells extracts were prepared from the following cells HeLa carrying either the LUC or LMP control vector, shPRDX1, shAPE1, or both shPRDX1 and shAPE1 and probed by Western blot using antibodies against APE1 and PRDX1. Anti-ACTβ antibody was used to monitor equal loading of the total extracts. The data are representative of two independent experiments. (**c**) Survival of cells treated with increasing concentrations of H_2_O_2_. The HeLa cell lines carrying the empty vector LMP or Luc or knockdown for either PRDX1, APE1 or both were treated with H_2_O_2_ for 1 h and scored for survivors after 10 days using the clongenic assay. The data are the average of three independent analyses.

PRDX1 exists in the nucleus, but the majority of the protein is localized to the cytosol of cells^26^. The deficiency of PRDX1 has been shown to cause elevated levels of oxidative damage to the genome^33^,^36^, and since APE1 is a key enzyme in the repair of these lesions, we tested whether shPRDX1 knockdown would impair the AP endonuclease function of APE1. In this experiment, we used a 42-mer double stranded oligonucleotide substrate containing a single uracil at position 21 that generates a 20-mer product following the action of uracil DNA glycosylase and cleavage of the resulting AP site by APE1^37^. Nuclear extracts prepared from HeLa cells did not show any significant differences in the level of AP endonuclease activity between the LMP control or when PRDX1 level was diminished by the shRNA (Fig. 4a,b lane 6 vs. 5). In a similar manner, we checked whether downregulating PRDX1 would have an effect on the AP endonuclease activity in cells with diminished levels of APE1. To test this, we first designed a system using the pSIREN-Zs-Green vector (LUC) to knockdown APE1 in the HeLa cells and then used the resulting clone shAPE1 (Fig. 4b, lane 3 vs.1) to downregulate PRDX1 using the LMP vector (Fig. 4b, lane 8 and 4). In these cells, the shRNA against APE1 downregulated APE1 level by nearly 75% (Fig. 4b, lane 3 or 4) and in the same cells the shRNA against PRDX1 downregulated PRDX1 by 80% (Fig. 4b, lane 8), as compared to the vector control (Fig. 4b, lanes 1 and 5). The assay revealed that shPRDX1 knockdown also did not further reduce the APE1 AP endonuclease activity in cells downregulated for APE1 (Fig. 4a), indicating that the diminished levels of PRDX1 has no effect at least on APE1 AP endonuclease activity. This finding was not specific to HeLa cells as similar results were obtained in HepG2 cells when PRDX1 was downregulated in the shAPE1 knockdown cells (Supplementary Fig. S2b–d).

Since PRDX1 can catalyze the detoxification of H_2_O_2_, we examined whether the downregulation of PRDX1 would sensitize cells to H_2_O_2_, and whether this would lead to increase damage to the genome requiring the 3′-repair diesterase function of APE1^4^. Interestingly, the downregulation of PRDX1 did not sensitize the HeLa cells (Fig. 4c), nor the HepG2 cells (Supplementary Fig. S2e), to increasing concentrations of H_2_O_2_. Likewise, the downregulation of PRDX1 did not further sensitize the shAPE1 knockdown HeLa cells when challenged with H_2_O_2_ (Fig. 4c). These findings exclude the possibility that PRDX1 plays a role in regulating APE1 DNA repair functions in processing H_2_O_2_-induced DNA lesions.

### PRDX1 downregulation stimulates IL-8 expression that depends on APE1 level

**Figure 5:**
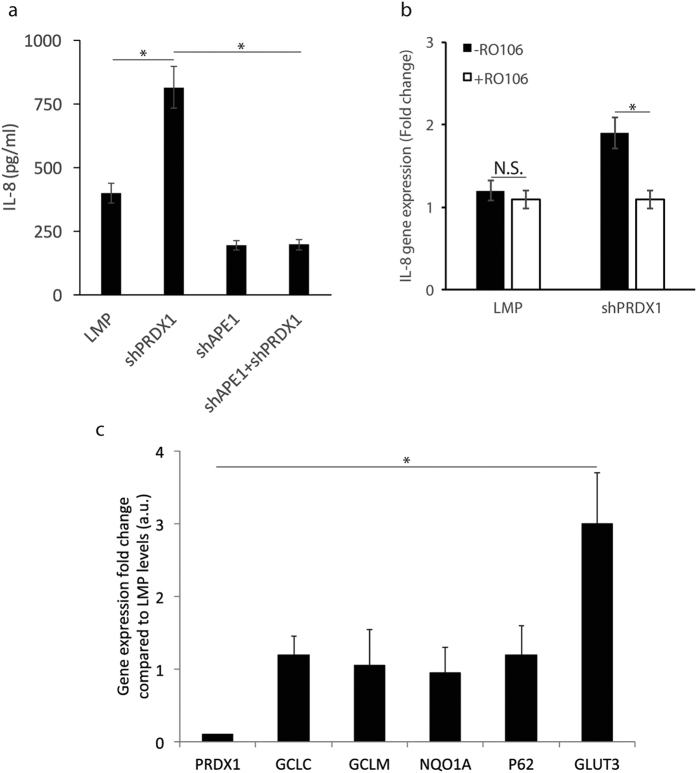
PRDX1 knockdown upregulates IL-8 in a manner requiring APE1 and NF-κB, but it has no effect on NRF2 targets. (**a**) PRDX1 knockdown induces IL-8 expression, but not in APE1 knockdown cells. The indicated HeLa cells were plated in 96-well plate at 15000 cells per well in duplicates in 200 μL complete DMEM with 10% FBS. The culture media was recovered and assay for IL-8 secretion using ELISA. The results are the averages of three independent experiments. (**b**) Inhibition of NF-κB activation prevents IL-8 gene induction in the PRDX1 knockdown cells. Total RNA was isolated from the HeLa cells carrying either the control LMP vector or shPRDX1 cells that were either untreated or treated with the NF-κB inhibitor RO106 (5 μM) and IL-8 gene expression was monitored by quantitative PCR analysis. The *ACTβ* gene was used for normalization. The results are the averages of two independent experiments. (**c**) NRF2 targets genes *GCLC*, *GCLM*, *NQO1A* and *P62* were unaffected in the PRDX1 knockdown cells. Total RNA was isolated from HeLa cells carrying either the control LMP vector or shPRDX1 cells and the expression of the indicated genes was monitored by quantitative PCR analysis. Gene expression was normalized to the *HPRT* gene and the levels in the shPRDX1 HeLa cells were expressed as fold-change relative to the levels in the LMP control, which was set at 1.0. The *GLUT3* gene was used as a positive control to monitor PRDX1 knockdown, as it was revealed to be highly inducible in these cells based on our microarray data. The results are the averages of two independent experiments.

A recent study documented that specific inhibition of the redox activity of APE1 by the inhibitor E3330 blocks TNF-α induced activation of the proinflammatory chemokine Interleukin-8 (IL-8) in HepG2 cells^38^. IL-8 can be regulated at the transcriptional level by several factors, including NF-κB, when cells encounter different stimuli such as chemical and environmental stresses^39^. Since APE1 can influence NF-κB-mediated gene expression, we examined whether disrupting PRDX1 interaction with APE1 would alter IL-8 production in the HeLa cells. Interestingly, when PRDX1 level was diminished, IL-8 secretion was stimulated by nearly 2-fold after 24 h of seeding the cells and analyzing the supernatant from the culture, as compared to the LMP control cells (Fig. 5a). This observation indicates that PRDX1 acts as a suppressor of IL-8 production in the cells.

We next tested if the stimulation of IL-8 production in the PRDX1 knockdown cells would depend on APE1 functional level. As such, we compared the level of IL-8 produced by the empty LMP vector cells, and by cells knockdown either for PRDX1, APE1 or both PRDX1 and APE1 (see Fig. 4b). Cells knockdown for APE1 displayed lower basal level of IL-8 production (Fig. 5a), consistent with the previous report that APE1 plays a role in IL-8 expression^38^. Importantly, the stimulated production of IL-8 caused by PRDX1 downregulation (Fig. 5a) was no longer observed in the cells that were also downregulated for APE1 (Fig. 5a). We interpret these data to indicate that PRDX1 association with APE1 may block APE1 redox function from turning on the transcription of IL-8.

### The stimulated production of IL-8 following PRDX1 downregulation depends on NF-κB activation

Since NF-κB is reported to activate the expression of IL-8^40^,^41^ and that APE1 can maintain this transcription factor in its active reduced state, we explored whether the mechanism by which the knockdown of PRDX1 led to the upregulation of IL-8 would involve NF-κB. To test this, we treated the LMP control and the corresponding shPRDX1 knockdown HeLa cells without and with the NF-κB inhibitor RO106 (5 μM), which inhibits IκBα ubiqutination, and thereby, prevents NF-κB translocation into the nucleus^42^. As shown in Fig. 5b, the NF-κB inhibitor blocked the activation of the *IL-8* gene in the cells knockdown for PRDX1 as quantified by the relative gene expression level using qPCR with ACTβ gene as the control. Thus, the mechanism by which PRDX1 knockdown stimulates *IL-8* gene expression requires NF-κB activation.

Besides positively regulating NF-κB, APE1 can also negatively influence the function of other transcription factors such as NRF2. The inhibition of APE1 redox activity has been shown to potently activate NRF2 target genes^12^. We checked whether PRDX1 knockdown would disrupt the redox state of APE1 and thus alter the expression levels of NRF2 targets. As shown in Fig. 5c, the shPRDX1 knockdown HeLa cells showed no significant changes in the expression levels of the established NRF2 target genes, such as *GLCC* and *GCLM*, relative to the levels from cells carrying the LMP control vector. In this analysis, the *GLUT3* gene, which we identified by microarray analysis to be negatively regulated by PRDX1, was used as a control to monitor its upregulation in the shPRDX1 knockdown cells (Fig. 5c, see discussion). Thus, even though downregulation of PRDX1 is known to increase the level of ROS in the cells^36^, this has no consequences on the expression of the NRF2 targets. In fact, it has been shown that the inhibition of APE1 redox activity leading to the activation of the NRF2 targets does not appear to occur via accumulation of ROS^12^. We therefore interpret our findings to suggest that in the PRDX1 knockdown cells APE1 remains redox active and poised to activate NF-κB.

### Low levels of PRDX1 mRNA correlate with poor survival in gastric cancer patients

**Figure 6:**
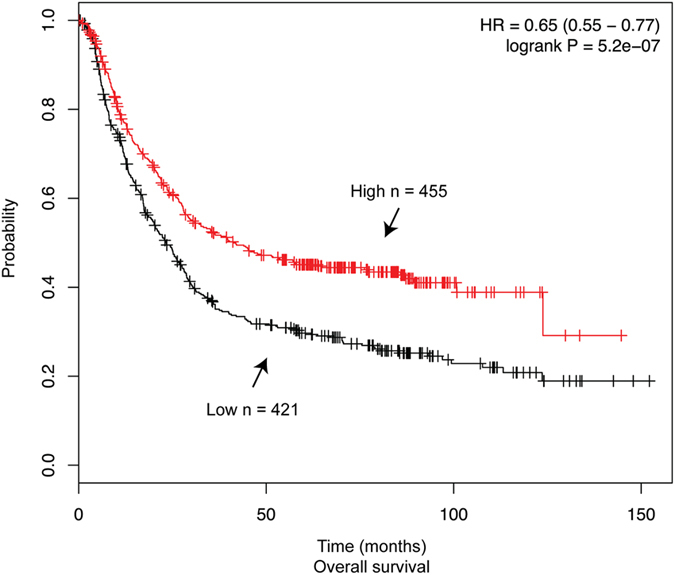
Low *PRDX1* mRNA level is associated with poor overall survival of patients with gastric cancer. Overall survival plot was generated online using the Kaplan-Meier Plotter based on signal intensity of the PRDX1 probe (208680_ at) in Affymetrix microarray gene expression data from gastric cancer patients of The Cancer Genome Atlas. Auto select best cut-off was chosen in the analysis and expression range of the probe was 2820–25698. A total of 876 patients were available and samples were split in two groups (high and low) according to the cutoff value. The hazard ratio with 95% confidence intervals and log rank *p* value was calculated.

We next checked whether *PRDX1* mRNA expression level would correlate with inflammation-associated cancers. Human gastric cancer is a leading cause of mortality worldwide and sporadic gastric tumors are the results of several factors, including diet and *Helicobacter pylori*-induced inflammation that elevate the production of ROS with the propensity to generate oxidatively damaged DNA^43^. To explore the potential significance of PRDX1 in preventing a proinflammatory response that would otherwise exacerbate the survival of gastric cancer patients, we used the Affymetrix gene expression to analyze 876 cases of gastric cancers from The Cancer Genome Atlas (TCGA) dataset^44^. Using the online tool Kaplan-Meier Plotter^45^,^46^, we observed that patients with a low level of *PRDX1* mRNA have significantly decreased overall survival (logrank *P* = 5.2e−07) than those with high expression levels of this gene (Fig. 6). This difference accounts for an average of 25 months increased life expectancy for patients with higher *PRDX1* expression levels (Fig. 6). It is possible that gastric cancer patients expressing reduced levels of PRDX1 are associated with poor outcome due to the induction of a proinflammatory response.

## Discussion

In this study, we used a stringent tandem affinity approach combined with mass spectrometry to compare APE1 interactome in the nucleus and the cytosol under physiological condition and when cells are treated with the oxidant H_2_O_2_. We uncovered a novel interaction between APE1 and PRDX1, which existed in both the nuclear and cytosolic fractions. PRDX1 did not influence APE1 DNA repair AP endonuclease activity or APE1 ability to repair damaged DNA when HeLa or HepG2 cells were exposed to H_2_O_2_. Consistent with this observation, it was shown that liver and kidney cell extracts derived from *prdx1*^*-/-*^ null mice showed normal levels of three additional enzymes uracil DNA glycosylase, 8-oxoguanine DNA glycosylase, and endonuclease III homologue I of the BER pathway^36^. Interestingly, PRDX1 knockdown facilitated APE1 detection in the nucleus by the anti-APE1 monoclonal and polyclonal antibodies, although neither APE1 level nor its cellular distribution was altered. Since PRDX1 forms a conjugate with APE1 *in vivo* (Fig. 2), we suggest that PRDX1 knockdown consequently sets APE1 free such that its redox function concomitantly activate binding of the transcription factor NF-κB onto the promoter of target genes, such as *IL-8* and causing its upregulation. IL-8 is a pro-inflammatory chemokine that has been linked with cancer migration, invasion and metastasis^21^. We propose that PRDX1 interaction with APE1 represents a novel mechanism, whereby APE1 redox function is engaged in a cycle of maintaining a pool of reduced PRDX1 to detoxify endogenously accumulated H_2_O_2_, and in this manner APE1 is not fully available to reduce transcription factors and activate gene expression. Disrupting this balance, for example, depleting PRDX1, is expected to augment pro-inflammatory response leading to cancer invasion and metastasis. In fact, it has been shown that mice lacking PRDX1 are viable, but develop several types of diseases including a high incidence of lymphomas and hepatocellular carcinomas^47^. Tissues from *prdx1*^-/-^ null mice display higher levels of ROS that correlated with significant accumulation of ROS-induced DNA base lesions as detected by liquid- and gas-chromatography/mass spectroscopy^36^. These oxidized DNA base lesions include the hydroxylated purines 8-hydroxy-2′-deoxyguanosine and 8-hydroxy-2′ deoxyadenosine and the cyclic nucleosides (5′R)-cyclo-2′-deoxyadenosine, (5′S)-cyclo-2′-deoxyadenosine, (5′R)-cyclo-2′-deoxyguanosine and (5′S)-cyclo-2′-deoxyguanosine, which can block transcription and replication^36^. APE1 can stimulate the DNA glycosylase OGG1 to repair 8-hydroxy-2′-deoxyguanosine^48^. Moreover, APE1 has been shown to possess a nucleotide incision repair activity that can directly nick the 5′-side of a damaged nucleotide and then converts the nick into a gap by its 3′ to 5′-exonuclease activity^49^,^50^. Indeed, it has been shown that APE1 can remove the (5′S)-cyclo-2′-deoxyadenosine when present at the 3′-termini of DNA^50^. Whether the loss of PRDX1 compromises APE1 ability to process these oxidized base lesions *in vivo*, for example, incising the lesions in an uncontrollable manner or unable to act on the lesions is not known. In any case, the unrepaired oxidatively produced lesions are likely to stall replication leading to DNA double strand breaks, which has the propensity to cause chromosomal rearrangements and translocations and thus also contribute to the elevated levels of age-related cancers in the *prdx1*^-/-^ null mice^47^.

**Figure 7:**
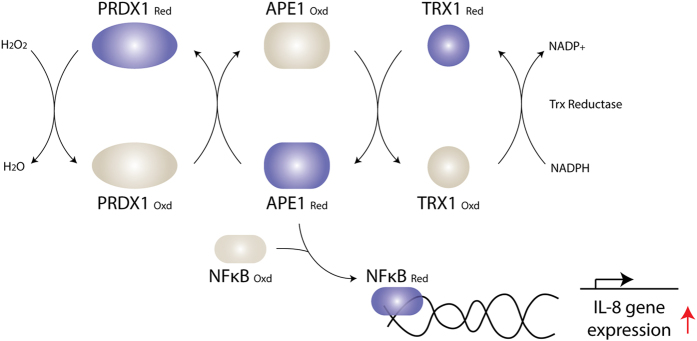
A model illustrating the propose role of APE1-PRDX1 interaction in regulating gene expression. Under normal metabolism, the reduced form of PRDX1_Red_ decomposes the endogenously produced H_2_O_2_ to water and in turn becomes oxidized. The oxidized PRDX1_Oxd_ is regenerated to the reduced form by the interaction with the reduced form of APE1 _Red_. In this scheme, APE1_Oxd_ is believed to be regenerated by thioredoxin I (TRX1). In the absence of PRDX1, there is a built up of H_2_O_2_ in the cells leading to the oxidation of NF-κB, which is set free from its inhibitor IKK and translocates to the nucleus. The oxidized NF-κB_Oxd_ has a redox cysteine (Cys62) that must be reduced in order for this transcription factor to bind to the promoter sequence of target genes such as IL-8. In the PRDX1 knockdown cells, APE1 is poised to reduce NF-κB resulting in the induction of IL-8 gene expression.

The observation that PRDX1, APE1 and NF-κB are involved in the regulation of IL-8, strongly suggests that a similar mechanism might be operational for limiting the expression of a number of oxidative stress response genes. We identified from a microarray analysis at least 19 additional genes that are upregulated in PRDX1 knockdown and are also regulated by APE1, such as PLA2G2A and FTHL19 involved in plasma membrane lipoprotein remodeling and iron homeostasis, respectively, under oxidative stress (Supplementary Fig. S4)^51^,^52^. These genes have in the common, at the promoter region, a binding site for NF-κB. Thus, it seems that the elevated level of ROS produced in the absence of PRDX1 may cause the remodeling of gene expression to produce proteins to combat the consequences of oxidative damage. We propose a model (Fig. 7) suggesting that under normal growth conditions, PRDX1 acts as an anti-inflammatory protein by sequestering a pool of reduced APE in the nucleus. This limits the availability of reduced APE1 to activate the oxidized form of NF-κB and thereby suppressing the induction of the pro-inflammatory target genes such as *IL-8* (Fig. 7). In support of this model, PRDX1 has been shown to interact with the Myc Box II region of c-Myc and alters its transcriptional function^53^. In the absence of PRDX1, c-Myc fails to properly regulate some of its target genes such as *cyclin B1*, *ODC*, *GAS1* and *GADD45*^36^.

A flood gate hypothesis has been proposed for the function of PRDX1 in combating oxidative stress^54^. In this simple model, it has been proposed that under the normal physiological redox state of the cell (perhaps low concentration of H_2_O_2_), the monomeric form of PRDX1 that possesses peroxidase activity deals with the burden of endogenously produced H_2_O_2_ as part of a first line defense that keeps NF-κB from being activated^55^. We provide evidence that once the level of PRDX1 is diminished the elevated level of the endogenous free radicals causes the activation of NF-κB, which is set free from its inhibitor subunit IκBα. The activated NF-κB then translocates to the nucleus leading to the induction of IL-8^36^,^40^,^41^,^56^. We showed that blocking the NF-κB translocation step with a known specific inhibitor RO106 prevented the induction of IL-8 in the PRDX1 knockdown cells. Binding of NF-κB to the promoter element of target genes such as *IL-8* requires that its oxidation sensitive cysteine Cys62 must be reduced. We believe that in the absence of PRDX1, APE1 is poised to maintain NF-κB in the reduced form such that it activates IL-8 expression. This is the most likely possibility as APE1 has been shown to reduce NF-κB^7^. Altogether, our data fit a model that downregulation of PRDX1 led to the activation of IL-8 in an APE1-dependent manner (see Fig. 7). Consistent with this model, APE1 knockdown blocked the high level expression of IL-8 in the PRDX1 knockdown cells.

It is noteworthy that while our studies were in progress, an independent group documented that PRDX1 depletion in the colon cancer cell line SW480 enhanced the production of ROS, and increased the expression levels of pro-inflammatory cytokines such as the tumor necrosis factor-α and interleukin (IL)-1β, as well as the chemokines IL-8 and CXCL1, and that this induction occurs by partial activation of NF-κB^57^. These independent observations reinforce the notion that PRDX1 is important to suppress the pathogenesis of inflammation-associated cancers such as colorectal and gastric and prolong the survival of the patients. Thus, the high expression of PRDX1 associated with some types of cancers, such as we show herein for gastric cancer (Fig. 6) might be to reduce inflammation.

In addition to its peroxidase activity, PRDX1 performs a role as a chaperone by protecting proteins from oxidation-induced inactivation and degradation^54^,^56^,^58^. For example, PRDX1 interacts with PTEN and prevents oxidation-induced inhibition of its phosphatase activity, which can occur as a result of a disulfide bond that forms between the two cysteines Cys71 and Cys124 in the N-terminal phosphatase domain of PTEN upon oxidation with H_2_O_2_^59^. The phosphatase activity of PTEN can function to dephosphorylate the lipid phosphatidylinositol triphosphates, which is required to activate the serine/threonine kinase AKT that plays a central role in suppressing H-Ras and ErbB-2 mediated oncogenic transformation^31^. Similarly, PRDX1 has been shown to maintain proper growth factors-mediated signaling pathways in different cancer models^32^,^60^. So far, we have no evidence that PRDX1 could serve as a chaperone to protect APE1 from inactivation or degradation, as neither APE1 endonuclease activity nor its stability was affected when PRDX1 was downregulated. We propose that oxidized PRDX1 molecules generated endogenously associate with a pool of reduced APE1, and that this interaction would serve to replenish the reduced state of PRDX1 (Fig. 7). In support of this model, inhibition of APE1 redox function with the specific inhibitor E3330 caused the accumulation of PRDX1 in its dimeric form (Fig. 2), a consequence of overoxidation of the monomeric PRDX1. Thus, under normal conditions the cycle of oxidation-reduction of PRDX1 would diminish the availability of reduced APE1 to activate the oxidized NF-κB, unless PRDX1 is depleted and according to our model leads to IL-8 expression. In the cycle of oxidation-reduction of PRDX1, the production of oxidized APE1 is expected to be reduced by thioredoxin, as oxidized APE1 has been shown previously to be in complex with reduced thioredoxin^34^. It is unlikely that thioredoxin is directly involved in the reduction of PRDX1, otherwise the inhibition of APE1 would not lead to the accumulation of the oxidized dimeric form of PRDX1. Nevertheless, further experiments are needed to examine whether inhibition of APE1 would downregulate thioredoxin or whether the depletion of thioredoxin would generate the oxidized dimeric form of PRDX1.

It has been documented that PRDX1 forms mixed disulfide intermediates with ASK1, which is required for the activation of the mitogen-activated protein kinase pathway in response to peroxide^30^. A more recent study documented that another member of the peroxiredoxin family, PRDX2 transfers oxidizing equivalents through direct protein-protein contact onto the STAT3 transcriptional activator resulting in the formation of PRDX2-STAT3 disulfide linked conjugates^61^. This relay results in the inactivation of STAT3 function in the transcriptional activation from the serum-induced promoter, but rescued upon PRDX2 depletion^61^. In a similar manner, it is possible that such relay exists between PRDX1 and APE1 and that the observed APE-PRDX1 conjugate could perform additional functions besides rendering a fraction of APE1 inactive and preventing NF-κB mediated activation of *IL-8*.

Since IL-8 plays a key role in the inflammatory response^62^,^63^, its upregulation in the PRDX1 knockdown cells may serve to recruit neutrophils to repair damage tissues caused by ROS. Thus, IL-8 expression must be turned off, otherwise the prolong activation of the inflammatory response can lead to severe tissue damage^64^. To date, multiple pathways including MEK/ERK, ATM and NF-κB, have been shown to influence IL-8 expression^21^,^65^. The fact that NF-κB is involved in all aspects of the immune response as well as other biological pathways, and that its activation can be promoted by the redox function of APE1, it seems that PRDX1-APE1 mixed disulfide conjugates would constitute a mechanism to tightly regulate IL-8 expression to control the inflammatory response. Interestingly, a very recent study demonstrated that IL-8, which is upregulated by ATM action on NF-κB through the intrinsic oxidative stress that exists in metastatic cancer cells, is required to promote migration and invasion that support tumour progression^21^,^66^,^67^. Thus, the interaction of PRDX1with APE1 might serve as an auxiliary mechanism to dampen the activation of NF-κB caused by the high oxidative stress that occurs in cancer cells, thereby reducing IL-8 expression from promoting its pro-metastatic functions.

In short, the long term consequences of PRDX1 functional loss are (i) the accumulation of ROS-induced oxidized DNA base lesions that might not be efficiently repaired by APE1 leading to genomic instability, as under these conditions APE1 could be targeted for its redox role, and (ii) the induction of IL-8 that has the ability to stimulate cancer cells invasion and metastasis.

## Materials and Methods

### Cell lines and cell culture

HeLa, HEK293T and HepG2 cells were cultured in Dulbecco’s Modified Eagle Medium (DMEM) (Wisent Inc., Cat No. 319-005-CL) supplemented with 10% of fetal bovine serum (FBS) (Wisent Inc., Cat. No. 095150) and in the presence of the antibiotics penicillin (100 U/ml) and streptomycin (0.1 mg/ml) (Wisent Inc., Cat. No. 450-201-EL). Cells were incubated at 37 °C and 5% CO_2_. The HeLaS cells were cultured in MEM media supplemented with 10% FBS and the antibiotics as above.

### Antibodies and reagents

The antibodies used in this study were anti-APE1 rabbit mAb (Epitomics, Cat No. 2851-1), anti-APE1 pAb antibody (Novus Biologicals, Cat No. NB100-101), anti-PRDX1 mAb antibody (Novus Biologicals, Cat No. NBP1-95676), anti-PRDX1 rabbit pAb (Cell Signalling, Cat No. 8732S), rabbit anti-FLAG pAb (Sigma, Cat No. F7425), mouse anti-HA mAb (Sigma, Cat No. H3663), goat anti-mouse IgG pAb HRP conjugate (Enzo Life Sciences, Cat No. ADI-SAB-100), goat anti-rabbit IgG pAb HRP conjugate (Enzo Life Sciences, Cat No. ADI-SAB-300-J), goat anti-rabbit IgG-CFL647 (Santa Cruz Biotechnology, sc-362292), MitoTracker Red CMXRos (Life Technologies, Cat No. M-7512). Amico Ultra (Millipore, Cat No. UFC501024). The mouse anti- ACTβ monoclonal antibodies (Santa Cruz Biotechnology, sc-69879) was used for normalizing protein content in cell extract.

### Plasmid constructs

pOZ-FH-N contains a Kozak sequence, an initiation methionine, FLAG and HA tags. pOZN-APE1 were constructed by first amplifying the human APE1 from K562 cell cDNA by PCR with the primers APE1-Xho1-F1 and APE1-Not1-R1 (Supplementary Table S1), and then subcloned the fragment into pOZ-FH-N following *Xhol* and *Notl* digestion. To knockdown PRDX1, several constructs were made based on the MSCV-LTRmiR30-PIG (LMP) vector (Thermo Scientific) following the manufacturer’s instructions. These knockdown constructs are named prdx1C1-2, prdx1C2-1, prdx1C3-1 and prdx1C4-1 and were all sequenced for confirmation. The hairpin shRNA templates are as following, sense and antisense sequences are underscored.

C1(HP_7670)TGCTGTTGACAGTGAGCGACCAGATGGTCAGTTTAAAGATTAGTGAAGCCACAGATGTAATCTTTAAACTGACCATCTGGCTGCCTACTGCCTCGGA

C2(HP_647595)TGCTGTTGACAGTGAGCGACCAGATGGTCAGTTTAAAGATTAGTGAAGCCACAGATGTAATCTTTAAACTGACCATCTGGCTGCCTACTGCCTCGGA

C3(HP_142580)TGCTGTTGACAGTGAGCGACCTGTCTGACTACAAAGGAAATAGTGAAGCCACAGATGTATTTCCTTTGTAGTCAGACAGGCTGCCTACTGCCTCGGA

C4(HP_820446)TGCTGTTGACAGTGAGCGACCTGTCTGACTACAAAGGAAATAGTGAAGCCACAGATGTATTTCCTTTGTAGTCAGACAGGCTGCCTACTGCCTCGGA. To knockdown APE1, two constructs were made based on vector RNAi-Ready pSIREN-RetroQ-ZsGreen (Clontech Laboratories, Inc.) following the manufacturer’s instructions: pSIREN shAPE1 1-1 and pSIREN shAPE1 2-1. Oligos APE1-shRNA-UP1 and APE1-shRNA-DWN1 were used for pSIREN shAPE1 1-1 targeting 5′-TGACAAAGAGGCAGCAGGA-3′ in APE1; oligos APE1-shRNA-UP2 and APE1-shRNA-DWN2 were used for pSIREN shAPE1 2-1 targeting 5′-GTCTGGTACGACTGGAGTACC-3′. Oligo sequences are as following:

APE1-shRNA-UP1: 5′-gatccGTGACAAAGAGGCAGCAGGATTCAAGAGATCCTGCT GCCTCTTTGTCATTTTTTg-3′; APE1-shRNA-DWN1: 5′-aattcAAAAAATGACAAAGA GGCAGCAGGATCTCTTGAATCCTGCTGCCTCTTTGTCACg-3′; APE1-shRNA-UP2: 5′-gatccGTCTGGTACGACTGGAGTACCTTCAAGAGAGGTACTCCAGTCGTACCAGACTTTTTTg-3′; and APE1-shRNA-DWN2: 5′-aattcAAAAAAGTCTGGTACGACTGGAGTACCTCTCTTGAAGGTACTCCAGTCGTACCAGACg-3′. pOZN-prdx1 was constructed by first amplifying human Prdx1from K562 cell cDNA by PCR with the primers pOZN-FH-prdx1F (5′-GCCGGAGGACTCGAGatgtcttcaggaaatgctaaaattggg-3′) and POZN-FH-prdx1R (5′-TCAGTCACGATGCGGCCGCtcacttctgcttggagaaatattcttt-3′) and then subcloned into pOZ-FH-N following *Xho*I and *Not*I digestion.

### Retrovirus preparation and infection

HEK293T cells were plated in 10 cm tissue cell culture plates, 24 h prior to transfection. The following day, retroviral vectors were cotransfected with pVSV-G and pCL-Eco retrovirus packaging vector using calcium phosphate transfection method. Supernatants were collected 36 to 48 h after transfection, filtered through a 0.45 μm filter and used directly to infect target cell lines. To infect HeLaS, HeLa or HepG2 cells, the cells were plated onto 10 cm tissue cell culture plates one day prior to infection at 35% confluence. The day of transfection, the old media were removed and replaced with viral supernatants/fresh media mixture (1:1) supplemented with 0.4 μg/ml Polybrene^®^ (Sigma-Aldrich). After 24 h infection, the viral media was removed and cells were washed at least twice with 1 x PBS and fresh media was added. Cells were subjected to selection 48 h after infection.

### Cell proliferation and clonogenic assays

To determine the average rate of population doublings, HeLa-LMP, HepG2-LMP, HeLa-shPRDX1 and HepG2-shPRDX1 cells were plated into 10 cm diameter petri-dishes in duplicate with initial cell density of 1.6 × 10^6^ cells/dish. After the indicated intervals (0 to 48 h), cells were trypsized and counted using the Countess^®^ Automated Cell Counter (Life Technologies). The numbers were converted into population doublings according to the following formula: [log (No. of cells counted)-log (No. of cell plated)]/log(2)^68^.

For the clonogenic assay, the indicated cell lines were plated in 6 cm diameter plates 24 h prior to treatment with and without the indicated concentrations of H_2_O_2_ for 1 hr and the colony forming unit was performed as described previously^69^.

### Purification of APE1 complex from HeLaS cells

A stable HeLaS cell line expressing human APE1 with N-terminal FLAG and HA tags was generated by retroviral transduction by using a previously described procedure with some modifications^23^. Nuclear extracts were prepared from 10 L of these cells as follows. The cells were washed twice with cold 1 x PBS, resuspended in hypotonic buffer (20 mM Hepes (K+), pH 7.6, 10 mM KCl, 1.5 mM MgCl_2_, 0.2 mM PMSF, 0.5 mM benzamidine, and 1 μg/mL each of leupeptin, aprotinin, and pepstatin), and incubated on ice for 10 min. The cells were then disrupted with approximately 10 strokes of a Wheaton Dounce homogenizer (B pestle) on ice. The nuclei were pelleted for 5 min at 4 °C in a clinical centrifuge and washed once with the same volume of hypotonic buffer as the pellets, then resuspended in extraction buffer (20 mM Hepes (K+), pH 7.6, 0.42 M KCl, 1.5 mM MgCl_2_, 0.2 mM EDTA, 25% (v/v) glycerol, 0.2 mM PMSF, 0.5 mM benzamidine, and 1 μg/mL each of leupeptin, aprotinin, and pepstatin), and sonicated (30% amplitude) for 2 min on ice. Insoluble material was pelleted for 15 min at 13,000 rpm in a Sorvall SS-34 rotor at 4 °C, and the soluble extract was dialyzed overnight against dialysis buffer (20 mM Hepes (K+), pH 7.6, 100 mM KCl, 1.5 mM MgCl_2_, 0.2 mM EDTA, 0.2 mM PMSF, 0.5 mM benzamidine, and 1 μg/mL each of leupeptin, aprotinin, and pepstatin). The extract was subjected to centrifugation for 15 min at 13,000 rpm in a Sorvall SS-34 rotor at 4 °C to remove insoluble material, and then used for affinity purification of the APE1 complex. To prepare the cytoskeleton fraction, KCl concentration was adjusted to 100 mM KCl and insoluble material was pelleted for 15 min at 13,000 rpm in a Sorvall SS-34 rotor at 4 °C. The supernatants were used for affinity purification of the APE1 complex.

The affinity purification of APE1 was performed by sequential anti-FLAG and anti-HA resins using methods similar to those previously described with some modifications^23^. Nuclear extracts were combined with 200 μL of FLAG (M2) resin (Sigma) and incubated overnight at 4 °C. The beads were pelleted and washed twice in batch with wash buffer (20 mM Hepes (K+), pH 7.6, 100 mM KCl, 5 mM MgCl_2_, 0.2 mM EDTA, 0.05% (v/v) NP-40, 10% (v/v) glycerol, 0.2 mM PMSF, 0.5 mM benzamidine, and 1 μg/mL each of leupeptin, aprotinin, and pepstatin). The beads were then transferred to a 1.5 mL column (Bio-Rad) and washed with 10 mL of wash buffer. Bound proteins were eluted by three successive 1 h incubations of the beads with 200 μL (for each incubation) of FLAG Elution buffer (Wash buffer plus 0.36 mg/mL FLAG peptide (Bio-basic)). The FLAG eluates were pooled, combined with 100 μL of HA resin, and incubated overnight at 4 °C. The resin was then washed in 1.5 mL column (Bio-Rad) with 10 mL of wash buffer. Bound proteins were eluted by three successive 1 h incubations of the beads with 200 μL (for each incubation) of HA elution buffer (Wash buffer plus 0.2 mg/mL HA peptide (Bio-basic)). The HA eluates were pooled and concentrated into 100 μL by 20% (w/v) trichloroacetic acid (TCA) precipitation, and an aliquot (10 μL) was analyzed by SDS-polyacrylamide gel electrophoresis and silver staining. A second aliquot (10 μL) was used for determining the identity of all the proteins in the complex by the Taplin Biological Mass Spectrometry Facility (Harvard Medical School, Boston, USA), and the remaining 80 μL kept frozen at −80 °C.

### Co-immunoprecipitation of APE1 and Prdx1 from cell and nuclear extracts

For the pull down of PRDX1, HeLaS cells carrying either the empty vector pOZN or the pOZN-APE1 were grown in 10 cm plates, washed twice in 1 x PBS, resuspended in 500 μL Lysis buffer (20 mM Hepes (K+), pH 7.6, 0.3 M KCl, 1.5 mM MgCl_2_, 0.2 mM EDTA, 0.3% (v/v) NP-40, 0.2 mM PMSF, 0.5 mM benzamidine, and 1 μg/mL each of leupeptin, aprotinin, and pepstatin), and sonicated for 2 seconds. The insoluble material was pelleted for 15 min at 13,000 rpm in a microcentrifuge, and the soluble extract was diluted with 1 mL of Lysis buffer with 10% (v/v) glycerol but lacking KCl and NP-40. Insoluble material was pelleted, and the soluble lysate was added to 30 μl of FLAG (M2) beads (Sigma) after 50 μL aliquots were taken for input, following incubation for 4 h at 4 °C. The beads were pelleted and washed with 3 × 1 mL of wash buffer (20 mM Hepes (K+), pH 7.6, 0.1 M KCl, 1.5 mM MgCl_2_, 0.2 mM EDTA, 0.01% (v/v) NP-40, 10% (v/v) glycerol, 0.2 mM PMSF, 0.5 mM benzamidine, and 1 μg/mL each of leupeptin, aprotinin, and pepstatin). The bound proteins were eluted by elution buffer (10 mM Tris pH 7.9, 10 mM EDTA, 1% SDS), and the beads were removed by centrifugation at 2000 RPM for 1 min in a microcentrifuge. The supernatants with the eluted proteins were processed for Western blot analysis.

For the reciprocal pull down of APE1, nuclear extract was prepared from HeLa cells as previously described^70^. Briefly, the sample from the nuclear extract (900 μl in hypotonic buffer derived from 100 million cells) was precleared with 30 μl of protein A magnetic beads for 2 h to remove all non-specific interactions, then the extract was collected and mixed with another fresh 30 μl of protein A magnetic beads followed by the addition of 5 μg of anti-Prdx1 polyclonal antibodies and the mixture was allowed to rotate slowly for overnight at 4 °C. The magnetic beads were collected, washed, and the total beads with bound proteins were resuspended in loading buffer for Western blot analysis.

### To detect APE1-PRDX1 conjugate

Typically 2 × 10^6^ HeLa cells (with and without 20 μM E3330 for 4 h) were incubated for 5 mins with 80 mM methyl methanethiosulfonate (Sigma) in PBS to block free thiols from disulfide exchange reactions. Cells were then resuspended in 1 ml of fresh hypotonic buffer (10 mM Tris pH 7.3, 10 mM KCL and 1.5 mM MgCl_2_) and transfered to a cold dounce homogenizer to be stroked 10 to 30 times to release the nuclei. Nuclei suspension was then spun down at 3900 rpm in an Eppendorf centrifuge at 4 °C for 15 mins. The nuclear pellet was resuspended in three volume of buffer II (50 mM Tris pH 7.3, 5 mM EDTA, 0.5% NP-40 and 300 mM NaCl) and incubated for 30 mins on ice. The sample was spun at 14,000 rpm in an Eppendorf centrifuge at 4 °C for 20 mins and the supernatant (nuclear extract) was transferred to a new tube. An equal volume of buffer O (50 mM Tris pH 7.3, 5 mM EDTA and 0.5% NP-40) was added to the nuclear extract. All buffers contained 10 mM beta-mercaptoethanol, 1 mM PMSF and complete protease inhibitor cocktail Tablets (Roche).

### Gel filtration

Post-nuclear supernatants were loaded on Fast Protein Liquid Chromatography (FPLC) ÄKTA purifier 10/100 system was used to co-elute interacting proteins under isocratic mode. Buffer solutions were degassed prior to use. Samples were filtered and centrifuged to remove debris that may clog the column. Small amount of sample (~0.5 mL) was loaded onto the Superose^TM^ 6 column equipped with ÄKTA purifier 10/100 FPLC system. Buffer A (containing 20 mM Hepes (K + ) pH 7.6, 100 mM KCl, 1.5 mM MgCl_2_, 0.2 mM EDTA, 0.2 mM PMSF, 0.5 mM benzamidine, and with 1 μg/mL each of leupeptin, aprotinin, and pepstatin) was pumped at flow rate of 0.25 ml/min. Before loading sample onto the column, the column was first equilibrated with 1 column volume of Buffer A and then samples were eluted with 2 column volumes of same buffer. All eluents were collected at 0.5 ml/fractions. Each fraction was concentrated by Amicon Ultra prior to the Western blot analysis.

### AP endonuclease assay

This assay was performed as previously described using a 42-mer substrate U21•G containing a single AP site^37^. Total cell extracts were incubated with the substrate and the reaction products were separated on 10% (w/v) polyacrylamide/7M urea gels and developed by autoradiography. The radiolabeled fragments were visualized by using a PhosphorImager (Storm 840, Molecular Dynamics), and ImageQuant LAS 400 was used for quantification. The intensity of the product was quantified using ImageJ and plotted as a percentage of increasing concentration of the total cell extracts.

### Immunofluorescence assay

For immunofluorescence assay, cells were grown on sterile cover slips placed in 24 well plates one day before treatment. The following day, after treatment for the indicated time interval, cells were washed with 1X PBS twice, and fixed for 30 min with 4% PFA containing 0.1% Triton X-100. The cells were then washed three times with PBS, each for 5 min, and incubated with the anti-APE1 primary antibody (1:2000) in PBS containing 5% FBS (fetal bovine serum) for 1 hr at room temperature or overnight at 4 °C. After the unbound primary antibodies were gently washed off thrice with 1X PBS for 5 min each, the cells were incubated with appropriate goat anti-rabbit IgG-CFL647 secondary antibodies (1:500) in PBS containing 5% FBS for 1 hr at 37 °C in dark. After 1 hr incubation at room temperature, the slides were counterstained with DAPI combined with 20 μl Vecta shield mounting solution (Vector Laboratories, Inc., CA). The cover slips with the cells were mounted facing down on the glass slides. Excess liquid was wiped out with filter paper. Finally the cover slips were sealed with nail polish. The cells were then visualized under the Zeiss Microscope with an appropriate barrier and excitation filters for FITC, DAPI and Cy5 visualization. Images were captured with AxioCam MRm using a 63x objective lens.

### Flow cytometry of APE1 Immunofluorescence

HeLa cells (WT, LMP, shPrdx1) were culture in 6-well plates until they reach almost full confluence. The cells were harvested with 1 ml trypsin 0.25%, centrifuged and the trypsin was removed. Cells were resuspended in 200 μl of PBS containing 4% paraformaldehyde (PFA) and incubated 15 min at room temperature (RT). Cells were centrifuged and PFA solution removed. Cells were resuspended in 50 μl of cold PBS and 450 μl of ice-cold methanol was added slowly followed by storage at −20 °C for at least 30 min. The cells were washed twice in PBS with 1% BSA, then resuspended in 150 μl of PBS with 1% BSA and incubated at RT for 20 min. Anti-APE1 antibody (1.5 μl) (Cell Signaling Technology, CAT # 4128) was added to the cells to be stained (the antibody was not added to a control set of cells). Cells were incubated 1 h RT with agitation and washed twice with 200 μl PBS with 1% BSA. The cells were resuspended in 500 μl PBS with 1% BSA and one drop of secondary Alexa Fluor^®^ 594 antibody (Thermo Fisher Scientific, CAT # R37117) was added as well as to the set of control cells. The cells were incubated 45 min at RT, centrifuged, and resuspended in 100 μl PBS. The cells were diluted in 400 μl in FACS tubes (BD Falcon 352052) then past through FACS Calibur. Alexa Fluor^®^ 594 was detected with FL2 (585/42). The threshold was determined using cells that were not incubated with the primary APE1 antibody and the fluorescence intensity of Alexa Fluor^®^ 594 was multiplied by the percentage of cells that are Alexa Fluor^®^ 594 positive.

### IL-8 quantification by ELISA

Human IL-8 ELISA kit (BD Biosciences, Cat. No. 555244) was used to quantify IL-8 in culture supernatants according to the manufacturer suggested protocol. Briefly 100 μL diluted capture antibody was added to each well of 96-well plates and incubated overnight at 4 °C. The following day, the unbound capture antibody was washed thrice with 300 μL 1X wash buffer (supplied with the kit) for 5 min each. The wells were blocked with 200 μL assay diluent for 1 hr at room temperature followed by three washes each time with 300 μL 1X washing buffer for 5 min each. Next 100 μL standard or samples were added to each well and the plate was incubated for 2 hr at RT followed by 5 times wash with 300 μL 1X washing buffer. 100 μL working 1X detection solution (Detection Ab+SAv-HRP at 1:250 dil) was added to each well and the plate was incubated for 1 hr at RT followed by 7 washes with 300 μL 1X washing buffer. Finally, 100 μL substrate solution was added to each well and incubated 30 mins at RT in dark. In order to stop the reaction, 50 μl stop solution was added to each well. All samples were set as duplicates and the absorbance was measured at 450 nm using a microplate reader (BioTek, EL800 Systems).

### Real-time PCR for gene expression quantification

For the isolation of total cellular RNA, the RNAeasy mini kit (Qiagen; Cat no. 74104) was used according to the manufacturer’s protocol. RNA concentration was measured with NanoDrop 2000 spectrophotometer (Thermo Scientific, Wilmington, Delaware, USA). The first-strand cDNA synthesis containing 100 ng of total RNA was primed with oligo(dT) using native avian myeloblastosis virus reverse transcriptase in the presence of RNase inhibitor RNaseOUT (Thermofisher Scientific; Cat no. 10777-019). The cDNA was diluted to 10 ng/μl using RNase-free water. Quantitative real-time PCR (qRT-PCR) amplification reactions were performed with the Applied Biosystems 7500 RT PCR system (Applied Biosystems), using the PerfeCTa SYBR Green SuperMix Low Rox PCR Kit (Quanta Biosciences; Cat no. 95056). The PCR reaction contained 10.0 μl of 2X SYBR Green SuperMix, 6.00 μl RNase-free water, 500 μM forward and reverse primers (the sequences of which are listed in Supp Table S1), and 8 ng cDNA, in a total volume of 20 μl. All reactions were run in triplicate with the following program: 95 °C for 3 min, followed by 45 cycles of 95 °C for 15 sec, 58 °C for 30 sec, and 72 °C for 20 sec, finishing with a melt cycle consisting of stepwise increases in temperature from 72 °C to 99 °C. The threshold numbers (C_t_ values) were set within the exponential phase of the reaction and were used to calculate the relative expression for each gene normalized to either *ACTβ* (β-actin) or *HPRT* RNA in each sample. The ratio of gene expression in experimental samples compared with control *ACTβ* (β-actin) or *HPRT* samples was then calculated. The fold change for the indicated gene relative to the reference gene was calculated and plotted as bar graph with standard deviation.

### Kaplan-Meier plotter analysis

The prognostic value of the *PRDX1* gene in gastric cancer was analyzed using the online tool Kaplan-Meier Plotter (http:/kmplot.com/analysis/), a database that integrates gene expression data and clinical information of breast, ovarian, lung and gastric cancers^45^,^46^. Kaplan-Meier Plotter uses three versions of the Affymetrix HG-U133 datasets (with 22,277 probe sets in common), and clinical data from Gene Expression Omnibus (GEO) and The Cancer Genome Atlas (TCGA) datasets. The expression of *PRDX1* in the TCGA dataset for gastric cancer was verified with the best specific probe (JetSet probes) of this gene (208680_ at). A total of 876 patients were available for analysis on overall survival. Patient samples were split into two groups according to the median value, using the query parameter of auto-select best cutoff. The signal range of the *PRDX1* probe was 2820–25698. The two patient groups (high and low expression levels) were compared using a Kaplan-Meier survival plot. The hazard ratio with 95% confidence intervals and log rank *p* value was calculated, and significance was set at *p* < 0.05.

### Microarray analysis

Microarray analysis was performed on cells carrying either the empty vector or the shRNA PRDX1 knockdown construct using Illumina BeadChip^®^ Microarray at Genome Québec Innovation Centre (McGill University, Montreal, Canada). The Illumina raw data files were imported into the FlexArray (ver 1.6.3) followed by execution of robust multi-array average (Lumi), which performs the background correction and Robust spline normalization. Principal components analysis (PCA) was used as tool to perform quality control for the data. For subsequent statistical analysis we used the SAM (Significance analysis of microarray), an algorithm implemented in Flexarray. Genes up-regulated (≥2 fold change) and down-regulated (≤0.5 fold change) with high statistical significance (*p* value < 0.01) were exported as text file for further analysis. Volcano plots of the differentially expressed genes were generated using –Log_10_ (*p* value) and Log_2_ (fold change). Venny 2.0.2 (Computational Genomic Services, CSIC) was employed for comparing gene list and drawing Venn’s diagram. Functional annotation clustering of the differentially expressed genes related to biological pathways were then performed using Gene Ontology (GO) term enrichment analysis and KEGG pathway mapping through DAVID Bioinformatics Resources 6.7 (http://david.abcc.ncifcrf.gov) with ease score = 0.01 and similarity threshold = 0.50. Gene Ontology (GO) terms significantly represented among differentially expressed genes were then listed with their corresponding *p* value and FDR in the Supplementary Table.

### Statistical analyses

Statistical differences were calculated by the unpaired two-tail t-test using the GraphPad Prism Statistical Software Mac Version 6 (*P < 0.01; **P < 0.001; N.S. non significant) and represented as ± S.D.

## Additional Information

**How to cite this article**: Nassour, H. *et al*. Peroxiredoxin 1 interacts with and blocks the redox factor APE1 from activating interleukin-8 expression. *Sci. Rep.* **6**, 29389; doi: 10.1038/srep29389 (2016).

## Supplementary Material

Supplementary Information
