# Supplementary material for: Peroxiredoxin 1 interacts with and blocks the redox factor APE1 from activating interleukin-8 expression
Source: Sci Rep. 2016 Jul 8;6:29389. doi: 10.1038/srep29389 (PMC4937415; doi:10.1038/srep29389)
Supplement: Supplementary Information [file srep29389-s1.doc]

**Supplemental data**

**Peroxiredoxin 1 interacts with and blocks the redox factor APE1 from activating interleukin-8 expression**

Hassan Nassour, Zhiqiang Wang, Amine Saad, Arturo Papaluca, Nicolas Brosseau, El Bachir Affar, Moulay A. Alaoui-Jamali and Dindial Ramotar

## Table S1. List of primers used in this study

| Serial No. | Study | Primer Name | Primer sequence |
| --- | --- | --- | --- |
| 1 | PCR | APE1-Xho1-F1 | 5’-CGTTCGTACTCGAGATGCCGAAGCGTGGGAAAA AG-3’ |
| 2 | PCR | APE1-Not1-R1 | 5’-CTTTTCCTTTTGCGGCCGCTCACAGTGCTAGGTATAGGG-3’ |
| 3 | shRNA | APE1-shRNA-UP1 | 5'-gatccGTGACAAAGAGGCAGCAGGATTCAAGAGATC CTGCTGCCTCTTTGTCATTTTTTg-3' |
| 4 | shRNA | APE1-shRNA-DWN1 | 5'-aattcAAAAAATGACAAAGAGGCAGCAGGATCTCTT GAATCCTGCTGCCTCTTTGTCACg-3' |
| 5 | shRNA | APE1-shRNA-UP2 | 5'-gatccGTCTGGTACGACTGGAGTACCTTCAAGAGAG GTACTCCAGTCGTACCAGACTTTTTTg-3' |
| 6 | shRNA | APE1-shRNA-DWN2 | 5'-aattcAAAAAAGTCTGGTACGACTGGAGTACCTCTCT TGAAGGTACTCCAGTCGTACCAGACg-3' |
| 7 |  | pOZN-FH-prdx1F | 5’-GCCGGAGGACTCGAGatgtcttcaggaaatgctaaaattggg-3’ |
| 8 |  | pOZN-FH-prdx1R | 5’-TCAGTCACGATGCGGCCGCtcacttctgcttggagaaatattcttt-3’ |
| 9 | qPCR | IL-8-F | 5′-ATAAAAAGCCACCGGAGCAC-3′ |
| 10 | qPCR | IL-8-R | 5′-GGCCAGCTTGGAAGTCATGT-3′ |
| 11 | qPCR | β-actin-F | 5'-CATGTACGTTGCTATCCAGGC-3' |
| 12 | qPCR | β-actin-R | 5'-CTCCTTAATGTCACGCACGAT-3' |
| 13 | qPCR | PRDX1-F | 5'-CGTCTTGTTCTTGCCTGGTG-3' |
| 14 | qPCR | PRDX1-R | 5'-ACATCTTCCTATCAGCAGTCCC-3' |
| 15 | qPCR | APE1-F | 5'-CAATACTGGTCAGCTCCTTCG-3' |
| 16 | qPCR | APE1-R | 5'-CAAATTCAGCCACAATCACCC-3' |
| 17 | qPCR | GLUT3-F | 5'-AACCAGCTGGGCATCGTTGTTGG-3' |
| 18 | qPCR | GLUT3-R | 5'-GCCACAATAAACCAGGGAATGGG-3' |
| 19 | qPCR | GCLC-F | 5'-GGACAAGAATACACCATCTCCA-3' |
| 20 | qPCR | GCLC-R | 5'-ATACTGCAGGCTTGGAATGTC-3' |
| 21 | qPCR | GCLM-F | 5'-GGGAACCTGCTGAACTGG-3' |
| 22 | qPCR | GCLM-R | 5'-CTGGGTTGATTTGGGAACTC-3' |
| 23 | qPCR | NQO1A-F | 5'-GCTGGTTTGAGCGAGTGTTC-3' |
| 24 | qPCR | NQO1A-R | 5'-CTGCCTTCTTACTCCGGAAGG-3' |
| 25 | qPCR | P62-F | 5'-GTGGTAGGAACCCGCTACAA-3' |
| 26 | qPCR | P62-R | 5'-GAGAAGCCCTCAGACAGGTG-3' |

**Table S2. APE1 protein interactome in the cytosol and the nucleus**

|  | **Proteins interacting with APE1 in cytosol under normal condition** |
| --- | --- |
| Protein name | Functions |
| APEX nuclease 1 | Repairs oxidative DNA damages *in vitro*. May have a role in protection against cell lethality and suppression of mutations. Removes the blocking groups from the 3'-termini of the DNA strand breaks generated by ionizing radiations and bleomycin (318 aa) |
| VIM  Vimentin | Vimentins are class-III intermediate filaments found in various non-epithelial cells, especially mesenchymal cells (466 aa) |
| ACTA2  actin, alpha 2 | smooth muscle, aorta; Actins are highly conserved proteins that are involved in various types of cell motility and are ubiquitously expressed in all eukaryotic cells (By similarity) (377 aa) |
| ANXA1  annexin A1 | Calcium/phospholipid-binding protein which promotes membrane fusion and is involved in exocytosis. This protein regulates phospholipase A2 activity. It seems to bind from two to four calcium ions with high affinity (346 aa) |
| PRDX1  peroxiredoxin 1 | Involved in redox regulation of the cell. Reduces peroxides with reducing equivalents provided through the thioredoxin system but not from glutaredoxin. May play an important role in eliminating peroxides generated during metabolism. Might participate in the signaling cascades of growth factors and tumor necrosis factor-alpha by regulating the intracellular concentrations of H_2_O_2_. Reduces an intramolecular disulfide bond in GDPD5 that gates the ability to GDPD5 to drive postmitotic motor neuron differentiation (By similarity) (199 aa) |
| ARF1  ADP-ribosylation factor 1 | GTP-binding protein that functions as an allosteric activator of the cholera toxin catalytic subunit, an ADP- ribosyltransferase. Involved in protein trafficking among different compartments. Modulates vesicle budding and uncoating within the Golgi complex. Deactivation induces the redistribution of the entire Golgi complex to the endoplasmic reticulum, suggesting a crucial role in protein trafficking. In its GTP-bound form, its triggers the association with coat proteins with the Golgi membrane. The hydrolysis of ARF1-bound GTP, which is mediated by ARFGAPs(181 aa) |
| PDIA6  protein disulfide isomerase family A, member 6 | May function as a chaperone that inhibits aggregation of misfolded proteins. Plays a role in platelet aggregation and activation by agonists such as convulxin, collagen and thrombin |
| PRDX3  peroxiredoxin 3 | Involved in redox regulation of the cell. Protects radical-sensitive enzymes from oxidative damage by a radical- generating system. Acts synergistically with MAP3K13 to regulate the activation of NF-kappa-B in the cytosol (256 aa) |
| HSP90B1  heat shock protein 90kDa beta (Grp94), member 1 | heat shock protein 90kDa beta (Grp94), member 1; Molecular chaperone that functions in the processing and transport of secreted proteins. Functions in endoplasmic reticulum associated degradation (ERAD). Has ATPase activity (803 aa) |
| PRDX2  peroxiredoxin 2 | Involved in redox regulation of the cell. Reduces peroxides with reducing equivalents provided through the thioredoxin system. It is not able to receive electrons from glutaredoxin. May play an important role in eliminating peroxides generated during metabolism. Might participate in the signaling cascades of growth factors and tumor necrosis factor-alpha by regulating the intracellular concentrations of H_2_O_2_ (198 aa) |
| HSPA5  heat shock 70kDa protein 5 | Probably plays a role in facilitating the assembly of multimeric protein complexes inside the ER (654 aa) |
| HSP90AB1  heat shock protein 90kDa alpha (cytosolic) | class B member 1; Molecular chaperone. Has ATPase activity (724 aa) |
| HSPD1  heat shock 60kDa protein 1 (chaperonin) | Implicated in mitochondrial protein import and macromolecular assembly. May facilitate the correct folding of imported proteins. May also prevent misfolding and promote the refolding and proper assembly of unfolded polypeptides generated under stress conditions in the mitochondrial matrix (573 aa) |
| ACTB  actin, beta | Actins are highly conserved proteins that are involved in various types of cell motility and are ubiquitously expressed in all eukaryotic cells (By similarity) (375 aa) |
| ACTBL2  actin, beta-like 2 | Actins are highly conserved proteins that are involved in various types of cell motility and are ubiquitously expressed in all eukaryotic cells (By similarity) (376 aa) |
|  | **Proteins interacting with APE1 in cytosol under hydrogen peroxide treatment** |
| EEF1A2  eukaryotic translation elongation factor 1 alpha 2 | This protein promotes the GTP-dependent binding of aminoacyl-tRNA to the A-site of ribosomes during protein biosynthesis (463 aa) |
| VIM vimentin | class-III intermediate filaments found in various non-epithelial cells, especially mesenchymal cells (466 aa) |
| ACTA2  actin, alpha 2 | smooth muscle, aorta; Actins are highly conserved proteins that are involved in various types of cell motility and are ubiquitously expressed in all eukaryotic cells (By similarity) (377 aa) |
| HSPA8  heat shock 70kDa protein 8 | Chaperone. Isoform 2 may function as an endogenous inhibitory regulator of HSC70 by competing the co-chaperones (646 aa) |
| TRAP1  TNF receptor-associated protein 1 | Chaperone that expresses an ATPase activity (704 aa) |
| HSPB1  heat shock 27kDa protein 1 | Involved in stress resistance and actin organization (205 aa) |
| PRDX1  peroxiredoxin 1 | Involved in redox regulation of the cell. Reduces peroxides with reducing equivalents provided through the thioredoxin system but not from glutaredoxin. May play an important role in eliminating peroxides generated during metabolism. Might participate in the signaling cascades of growth factors and tumor necrosis factor-alpha by regulating the intracellular concentrations of H_2_O_2_. Reduces an intramolecular disulfide bond in GDPD5 that gates the ability to GDPD5 to drive postmitotic motor neuron differentiation (By similarity) (199 aa) |
| PDIA6  protein disulfide isomerase family A member 6 | May function as a chaperone that inhibits aggregation of misfolded proteins. Plays a role in platelet aggregation and activation by agonists such as convulxin, collagen and thrombin |
| HSP90B1  heat shock protein 90kDa beta (Grp94), member 1 | Molecular chaperone that functions in the processing and transport of secreted proteins. Functions in endoplasmic reticulum associated degradation (ERAD). Has ATPase activity (803 aa) |
| TUBA1C  tubulin, alpha 1c | Tubulin is the major constituent of microtubules. It binds two moles of GTP, one at an exchangeable site on the beta chain and one at a non-exchangeable site on the alpha-chain (By similarity) (449 aa) |
| PKM2  pyruvate kinase | Glycolytic enzyme that catalyzes the transfer of a phosphoryl group from phosphoenolpyruvate (PEP) to ADP, generating ATP. Stimulates POU5F1-mediated transcriptional activation. Plays a general role in caspase independent cell death of tumor cells. The ratio betwween the highly active tetrameric form and nearly inactive dimeric form determines whether glucose carbons are channeled to biosynthetic processes or used for glycolytic ATP production. The transition between the 2 forms contributes to the control of glycolysis and is important for tumor cell proliferat (531 aa) |
| HSPA5  heat shock 70kDa protein 5 | Probably plays a role in facilitating the assembly of multimeric protein complexes inside the ER (654 aa) |
| HSP90AB1  heat shock protein 90kDa alpha (cytosolic) | Molecular chaperone. Has ATPase activity (724 aa) |
| P4HB  prolyl 4-hydroxylase, beta polypeptide | This multifunctional protein catalyzes the formation, breakage and rearrangement of disulfide bonds. At the cell surface, seems to act as a reductase that cleaves disulfide bonds of proteins attached to the cell. May therefore cause structural modifications of exofacial proteins. Inside the cell, seems to form/rearrange disulfide bonds of nascent proteins. At high concentrations, functions as a chaperone that inhibits aggregation of misfolded proteins. At low concentrations, facilitates aggregation (anti-chaperone activity). (508 aa) |
| EEF1A1  eukaryotic translation elongation factor 1 alpha-like 7 | This protein promotes the GTP-dependent binding of aminoacyl-tRNA to the A-site of ribosomes during protein biosynthesis (By similarity) (462 aa |
| HSP90AA1  heat shock protein 90kDa alpha (cytosolic) | class A member 1; Molecular chaperone. Has ATPase activity (By similarity) (854 aa) |
| HSPD1  heat shock 60kDa protein 1 (chaperonin) | Implicated in mitochondrial protein import and macromolecular assembly. May facilitate the correct folding of imported proteins. May also prevent misfolding and promote the refolding and proper assembly of unfolded polypeptides generated under stress conditions in the mitochondrial matrix (573 aa) |
| TUBB2C  tubulin, beta 2C | Tubulin is the major constituent of microtubules. It binds two moles of GTP, one at an exchangeable site on the beta chain and one at a non-exchangeable site on the alpha-chain (By similarity) (445 aa) |
| PRDX6  peroxiredoxin 6 | Involved in redox regulation of the cell. Can reduce H_2_O_2_ and short chain organic, fatty acid, and phospholipid hydroperoxides. May play a role in the regulation of phospholipid turnover as well as in protection against oxidative injury (224 aa) |
| ACTB  actin, beta | Actins are highly conserved proteins that are involved in various types of cell motility and are ubiquitously expressed in all eukaryotic cells (By similarity) (375 aa) |
| PHGDH | phosphoglycerate dehydrogenase (533 aa) |
| CPS1  carbamoyl-phosphate synthetase 1 | mitochondrial; Involved in the urea cycle of ureotelic animals where the enzyme plays an important role in removing excess ammonia from the cell (1506 aa) |
| ACTBL2  actin, beta-like 2 | Actins are highly conserved proteins that are involved in various types of cell motility and are ubiquitously expressed in all eukaryotic cells (By similarity) (376 aa) |
|  | **Proteins interacting with APE1 in nucleus under normal condition** |
| RPS19  ribosomal protein S19 | Required for pre-rRNA processing and maturation of 40S ribosomal subunits (145 aa) |
| ACTA2  actin, alpha 2 | smooth muscle, aorta; Actins are highly conserved proteins that are involved in various types of cell motility and are ubiquitously expressed in all eukaryotic cells (By similarity) (377 aa) |
| PRDX1  peroxiredoxin 1 | Involved in redox regulation of the cell. Reduces peroxides with reducing equivalents provided through the thioredoxin system but not from glutaredoxin. May play an important role in eliminating peroxides generated during metabolism. Might participate in the signaling cascades of growth factors and tumor necrosis factor-alpha by regulating the intracellular concentrations of H_2_O_2_. Reduces an intramolecular disulfide bond in GDPD5 that gates the ability to GDPD5 to drive postmitotic motor neuron differentiation (By similarity) (199 aa) |
| NPM1  nucleophosmin | Involved in diverse cellular processes such as ribosome biogenesis, centrosome duplication, protein chaperoning, histone assembly, cell proliferation, and regulation of tumor suppressors TP53/p53 and ARF. Binds ribosome presumably to drive ribosome nuclear export. Associated with nucleolar ribonucleoprotein structures and bind single-stranded nucleic acids. Acts as a chaperonin for the core histones H3, H2B and H4 (294 aa) |
| LMNA  lamin A/C | Lamins are components of the nuclear lamina, a fibrous layer on the nucleoplasmic side of the inner nuclear membrane, which is thought to provide a framework for the nuclear envelope and may also interact with chromatin. Lamin A and C are present in equal amounts in the lamina of mammals (664 aa) |
|  | **Proteins interacting with APE1 in nucleus under H_2_O_2_ treatment** |
| LMNA  lamin A/C | Lamins are components of the nuclear lamina, a fibrous layer on the nucleoplasmic side of the inner nuclear membrane, which is thought to provide a framework for the nuclear envelope and may also interact with chromatin. Lamin A and C are present in equal amounts in the lamina of mammals (664 aa) |

**Peroxiredoxin 1 interacts with and blocks the redox factor APE1 from activating interleukin-8 expression**

Hassan Nassour, Zhiqiang Wang, Amine Saad, Arturo Papaluca, Nicolas Brosseau, El Bachir Affar, Moulay A. Alaoui-Jamali and Dindial Ramotar

**Legends for Supplemental Figures**

**Figure S1.** APE1 nuclear and cytosolic interactomes. a, b, and c, APE1 complex following purification from nuclear and cytosolic extracts from HeLaS cells expressing FH-APE1 without and with treatment (panel c) with 25 µM H_2_O_2_ for 1 h, respectively. The nuclear and cytosolic fractions were subjected to tandem immunoprecipitation with anti-FLAG followed by anti-HA resins. APE1 complex were finally eluted by HA peptides and subjected to mass spectrometry to identify all the proteins forming part of the APE1 interactome. The proteins interactome were visualized by searching STRING software, a database of known and predicted protein interactions.

**Figure S2.** PRDX1 knockdown did not interfere with APE1 ability to repair damaged DNA in HepG2 cells. **a,** Western blot analysis showing PRDX1 knockdown in HepG2 cells. **b** and **c**, qPCR analysis showing the expression of the *APE1* and *PRDX1* genes, respectively, in the indicated HepG2 cell lines. The data is representative of two independent experiments. **d**, Total nuclear extracts derived from the four indicated HepG2 cell lines were assayed for APE1 AP endonuclease activity. Cleavage of the substrate produced a 20-mer product that was quantified and plotted against the concentration of the nuclear extracts. The AP endonuclease activity measurement is the average of two experiments. **e**, Survival of cells treated with increasing concentrations of H_2_O_2_. The HepG2 cell lines carrying the empty vector LMP or Luc or knockdown for either PRDX1, APE1 or both were treated with H_2_O_2_ for 1 h and scored for survivors after 10 days using the clongenic assay. The data is the average of three independent analyses.

**Figure S3.** Prdx1 knockdown augments APE1 detection in the nucleus as revealed by indirect immunofluorescence using monoclonal anti-APE1 antibody. HeLa-LMP and HeLa-shPRDX1 cells were stained with monoclonal anti-APE1 antibodies (see Materials and Methods). Images were captured with a Zeiss epifluorescent microscope using Cy5 and DAPI-UV filters at 67 X magnification, and then processed with ImageJ software. Merged images overlapped DAPI-stained nucleus (blue) with Cy5 staining of APE1 (red). FITC filter marks the cells expressing the GFP protein from the LMP vector.

**Figure S4**. Venn diagram showing 19 genes that are upregulated in PRDX1 knockdown cells and are regulated by APE1 at the transcriptional level.

**Peroxiredoxin 1 interacts and blocks the redox factor APE1 from activating interleukin-8 expression**

Hassan Nassour, Zhiqiang Wang, Amine Saad, Arturo Papaluca, Nicolas Brosseau, El Bachir Affar, Moulay A. Alaoui-Jamali and Dindial Ramotar


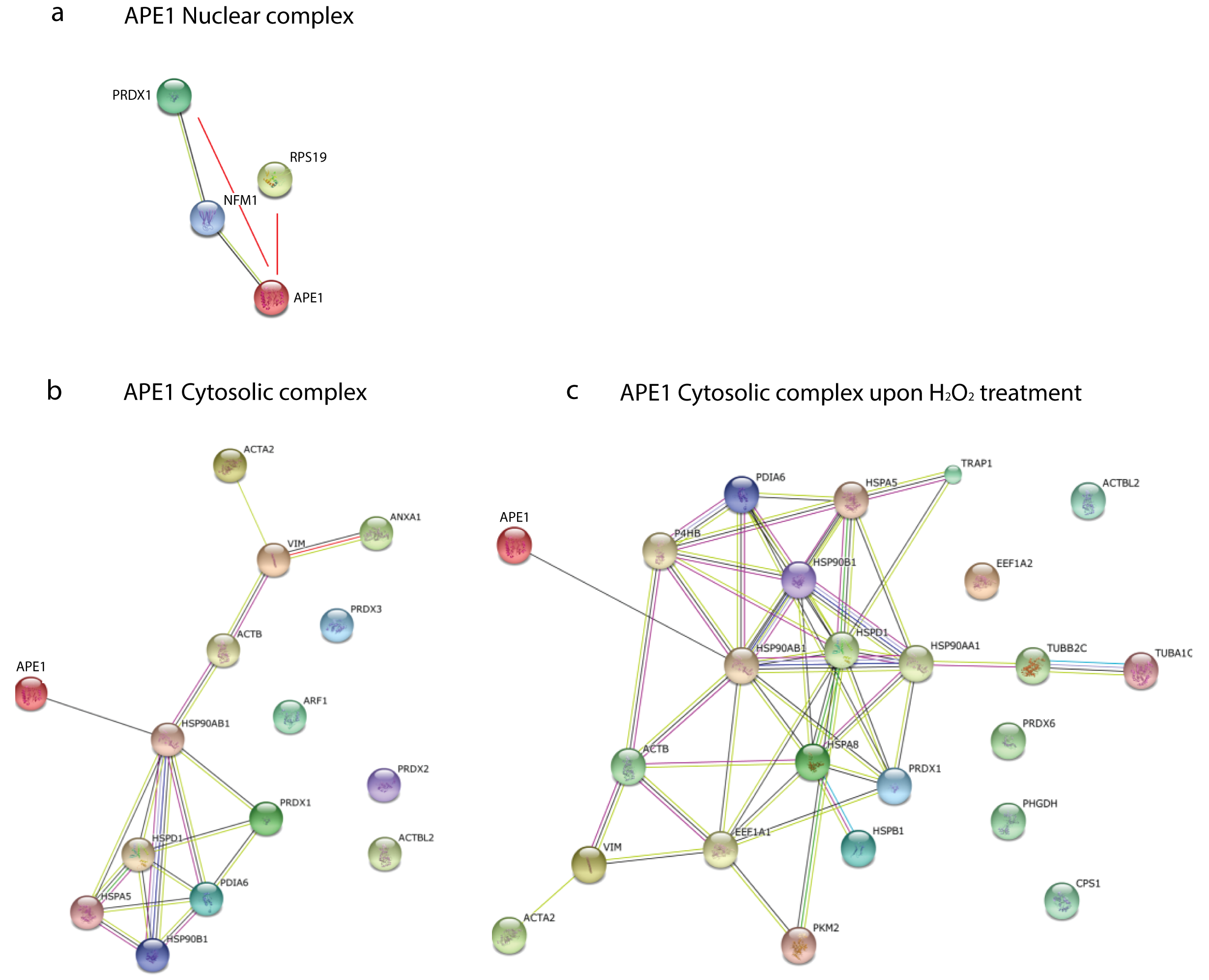


**Figure S1.** APE1 nuclear and cytosolic interactomes. a, b, and c, APE1 complex following purification from nuclear and cytosolic extracts from HeLaS cells expressing FH-APE1 without and with treatment (panel c) with 25 µM H_2_O_2_ for 1 h, respectively. The nuclear and cytosolic fractions were subjected to tandem immunoprecipitation with anti-FLAG followed by anti-HA resins. APE1 complex were finally eluted by HA peptides and subjected to mass spectrometry to identify all the proteins forming part of the APE1 interactome. The proteins interactome were visualized by searching STRING software, a database of known and predicted protein interactions.


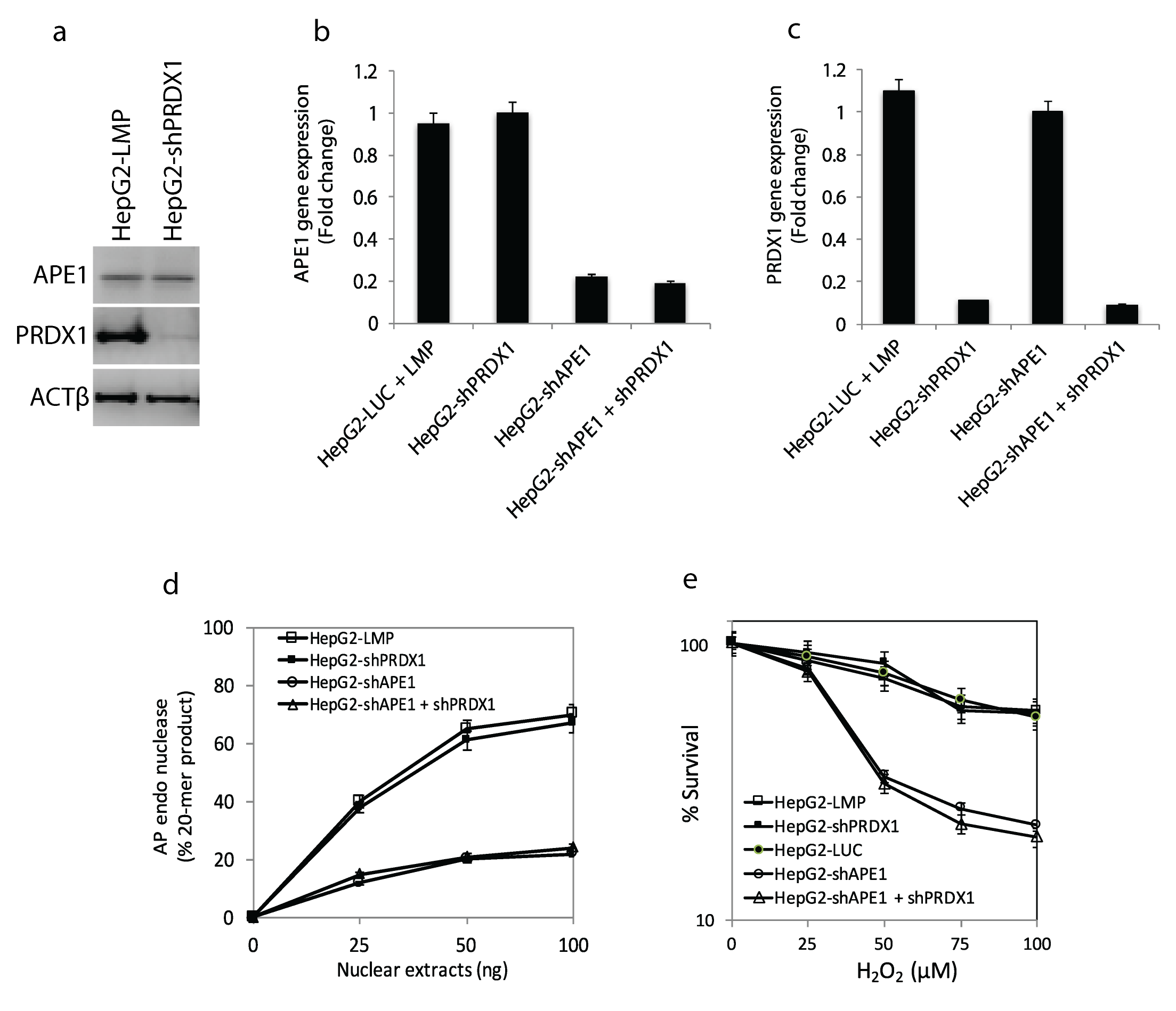


**Figure S2.** PRDX1 knockdown did not interfere with APE1 ability to repair damaged DNA in HepG2 cells. **a,** Western blot analysis showing PRDX1 knockdown in HepG2 cells. **b** and **c**, qPCR analysis showing the expression of the *APE1* and *PRDX1* genes, respectively, in the indicated HepG2 cell lines. The data is representative of two independent experiments. **d**, Total nuclear extracts derived from the four indicated HepG2 cell lines were assayed for APE1 AP endonuclease activity. Cleavage of the substrate produced a 20-mer product that was quantified and plotted against the concentration of the nuclear extracts. The AP endonuclease activity measurement is the average of two experiments. **e**, Survival of cells treated with increasing concentrations of H_2_O_2_. The HepG2 cell lines carrying the empty vector LMP or Luc or knockdown for either PRDX1, APE1 or both were treated with H_2_O_2_ for 1 h and scored for survivors after 10 days using the clongenic assay. The data is the average of three independent analyses.


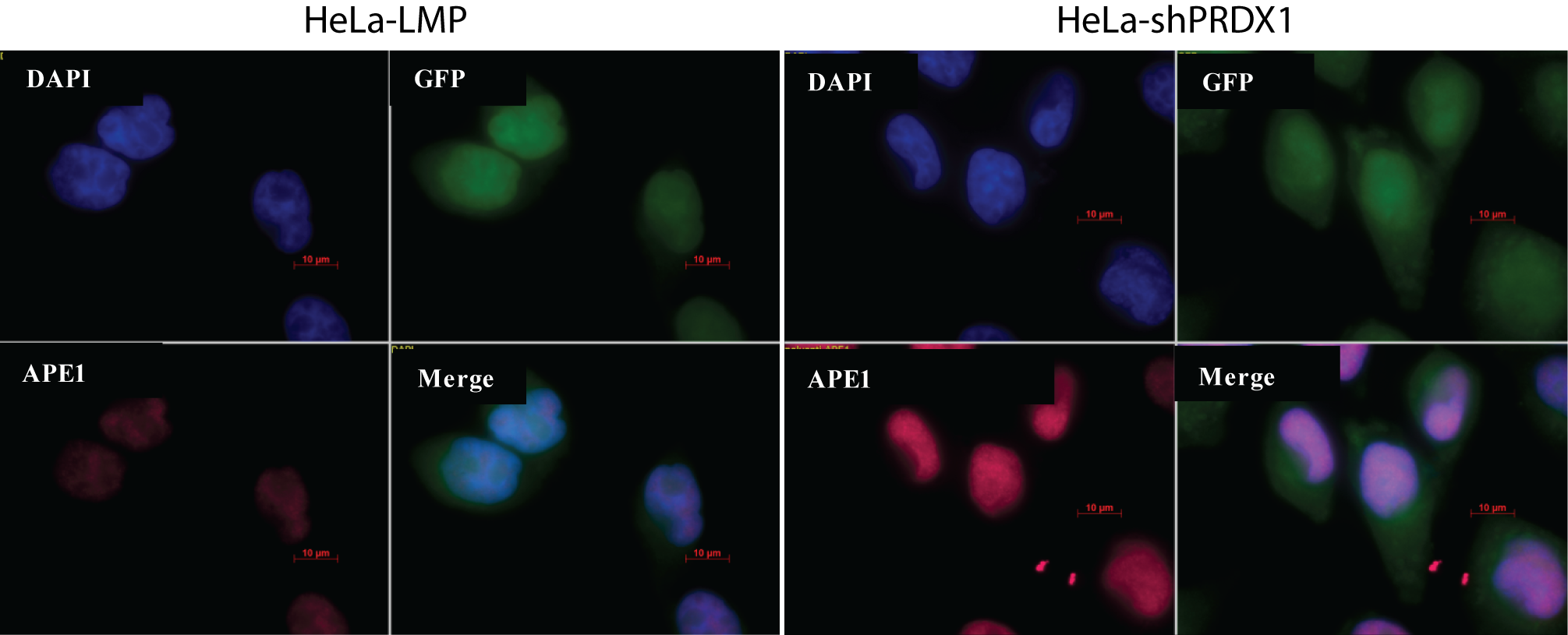


**Figure S3.** Prdx1 knockdown augments APE1 detection in the nucleus as revealed by indirect immunofluorescence using monoclonal anti-APE1 antibody. HeLa-LMP and HeLa-shPRDX1 cells were stained with monoclonal anti-APE1 antibodies (see Materials and Methods). Images were captured with a Zeiss epifluorescent microscope using Cy5 and DAPI-UV filters at 67 X magnification, and then processed with ImageJ software. Merged images overlapped DAPI-stained nucleus (blue) with Cy5 staining of APE1 (red). FITC filter marks the cells expressing the GFP protein from the LMP vector.


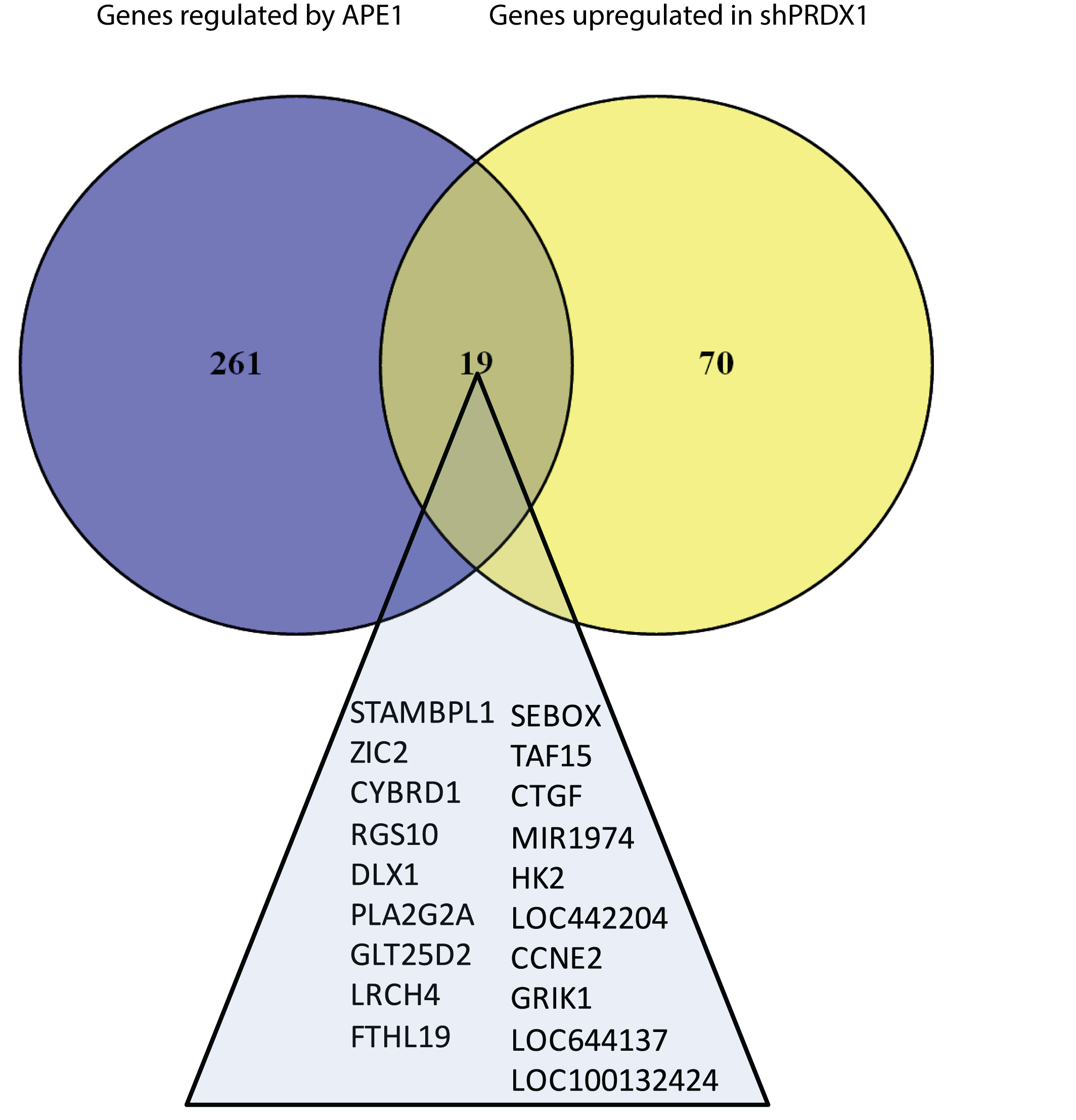


**Figure S4**. Venn diagram showing 19 genes that are upregulated in PRDX1 knockdown cells and are regulated by APE1 at the transcriptional level.
